# Substrate heterogeneity promotes cancer cell dissemination through interface roughening

**DOI:** 10.1126/sciadv.aed0587

**Published:** 2026-09-09

**Authors:** Zuzana Dunajová, Saren Tasciyan, Juraj Májek, Jack Merrin, Robert P. Jenkins, Erik Sahai, Michael Sixt, Edouard Hannezo

**Affiliations:** ^1^ Institute of Science and Technology Austria (IST Austria), am Campus 1, 3400 Klosterneuburg, Austria.; ^2^ Tumour Cell Biology Laboratory, The Francis Crick Institute, London, UK.

## Abstract

Although studies of tumor invasion have mainly emphasized the evolution of genetic and epigenetic heterogeneity, cellular behaviors also depend on the physical microenvironment. Yet, the tumor microenvironment is both complex and continuously remodeled, making it hard to rigorously test its causal influence on tumor dissemination. Here, we adopted a reductionist approach to studying tumor cell invasion in microengineered environments of tunable patterned geometries. We find that not only the presence of obstacles but more unexpectedly their spatial disorder causes a drastic shift from a collective to a single-cell mode of invasion—comparable in strength to loss of cell-cell adhesion. Combining live-imaging and perturbation experiments with minimal biophysical modeling, we demonstrate that cell dissemination results both from local geometrical constraints and a global integration of spatial disorder over time. We identify generic universality classes of invasion dynamics dependent on environmental geometry, enabling us to identify quantitative physical principles for tumor dissemination.

## INTRODUCTION

Cellular heterogeneity is at the core of tumor evolution. The canonical view suggests that new genetic variants produce new cancer cell phenotypes that invade extratumoral micromilieus. If such variants experience favorable conditions, they clonally expand and breed new heterogeneity. In an iterative process, the tumor ultimately reaches the fatal state of systemic dissemination (*1*–*3*). While heritable genetic variations have long been recognized to play a crucial role, phenotypic variation can also arise from epigenetic regulation or even result from transient signaling states (*4*, *5*). In any case, variation and selection of cancer cells are not autonomous to the tumor but occur through reciprocal cross-talk with the untransformed microenvironment (*6*–*8*). In some tumor models, invasiveness is not an intrinsic property of tumor cells but rather a transient response of rare cancer cells to signals released from a benign stroma (*9*–*11*). This raises the possibility that heterogeneous extratumoral microenvironments may act as drivers of malignancy (*12*), although this remains largely untested due to the experimental difficulty of controlling substrate heterogeneity.

In recent years, reductionist approaches have gained increasing attention, showing that cell behaviors can emerge from physical principles alone, without requiring any genetic or epigenetic changes. For instance, bottom-up reconstitution of microenvironments has been successfully used to model cellular responses (*13*) and cancer dissemination in response to specific local topographies of extracellular matrix (ECM) (*14*, *15*). Cellular collectives can also undergo fluid-solid transitions as a function of cell-cell and cell-ECM adhesion, cell density, or active migratory forces (*16*–*22*), with fluidized states favoring invasion (*23*–*27*). This fluidization can create large mechanical stresses on nuclei, triggering mechano-adaptative responses that feed back to further promote malignancy (*28*). In parallel, the interfaces of spreading tumor colonies display fractal-like features, which have been proposed to follow generic models for growing interfaces, such as the Kardar-Parisi-Zhang (KPZ) equation (*29*, *30*) or the molecular beam epitaxy equation (*31*), which have been validated in diverse nonliving systems (*32*, *33*). However, prior studies have almost exclusively explored migration in homogeneous or ordered environments, leaving the impact of extracellular heterogeneity unexplored.

Here, we combine reductionist microengineering pillar arrays with live cell imaging and minimal particle-based simulations to explore how environmental disorder shapes collective migration in 2.5D (hereafter referred to as “invasion”). We chose to specifically manipulate environmental geometry while leaving all other aspects, such as cell-cell and cell-matrix adhesions, constant. This minimal change demonstrated complex impacts on interfacial growth and tumor dispersion. In particular, we found that spatial disorder robustly enhances single-cell detachments from the invasive front, a phenomenon that emerges both from local features of substrate heterogeneity, as well as a much more global effect of temporal integration of the heterogeneity causing the roughening of the tumor front. Substrate heterogeneity fosters transition to a different universality class of invasion dynamics, which we characterize experimentally and theoretically. This led us to derive a quantitative description of the determinants of cellular dispersion, which we tested by experiments in different environments with structured geometries. Our results link microenvironmental heterogeneity to invasion dynamics through universal physical principles, providing a mechanistic basis for how disorder promotes cellular dispersion and, potentially, malignant progression.

## RESULTS

### Environmental mechanics and heterogeneity are sufficient to drive dispersion in vitro

As a cellular model for tumor invasion, we used A431 human carcinoma cells (*34*). This cell line is derived from a primary tumor of epithelial origin and forms stable cell-cell adhesions due to the expression of E- and P-cadherin (*35*). On fibronectin-coated two-dimensional (2D) surfaces, A431 cells spread radially as a cohesive front, without cellular detachments (fig. S1A). To test invasion into geometrically precisely controlled environments, we engineered microfluidic devices where a centrally applied pool of cells spreads into a fibronectin-coated confined space of 9-μm height. The two confining surfaces were intersected with arbitrarily positionable 9-μm-diameter pillars, creating a 3D chamber that forces cells to invade into the “pillar forest” as a monolayer. To ensure physiological relevance of our assays (*36*–*38*), we measured collagen pore size distribution in cervical squamous cell carcinoma (SCC) as well as in the dermis underlying the normal human cervical epithelium (fig. S1, B and C). On the basis of these parameters, we chose a 9-μm pillar spacing, which we also found to be adequate as cells could pass through by only moderately deforming their ∼12- to 18-μm-diameter nuclei. The setup allowed free medium exchange and long-term life imaging (>1 week) using genetically encoded fluorescent reporters for cytoplasm (mCherry) and nuclei [green fluorescent protein (GFP)].

**Fig. 1.**
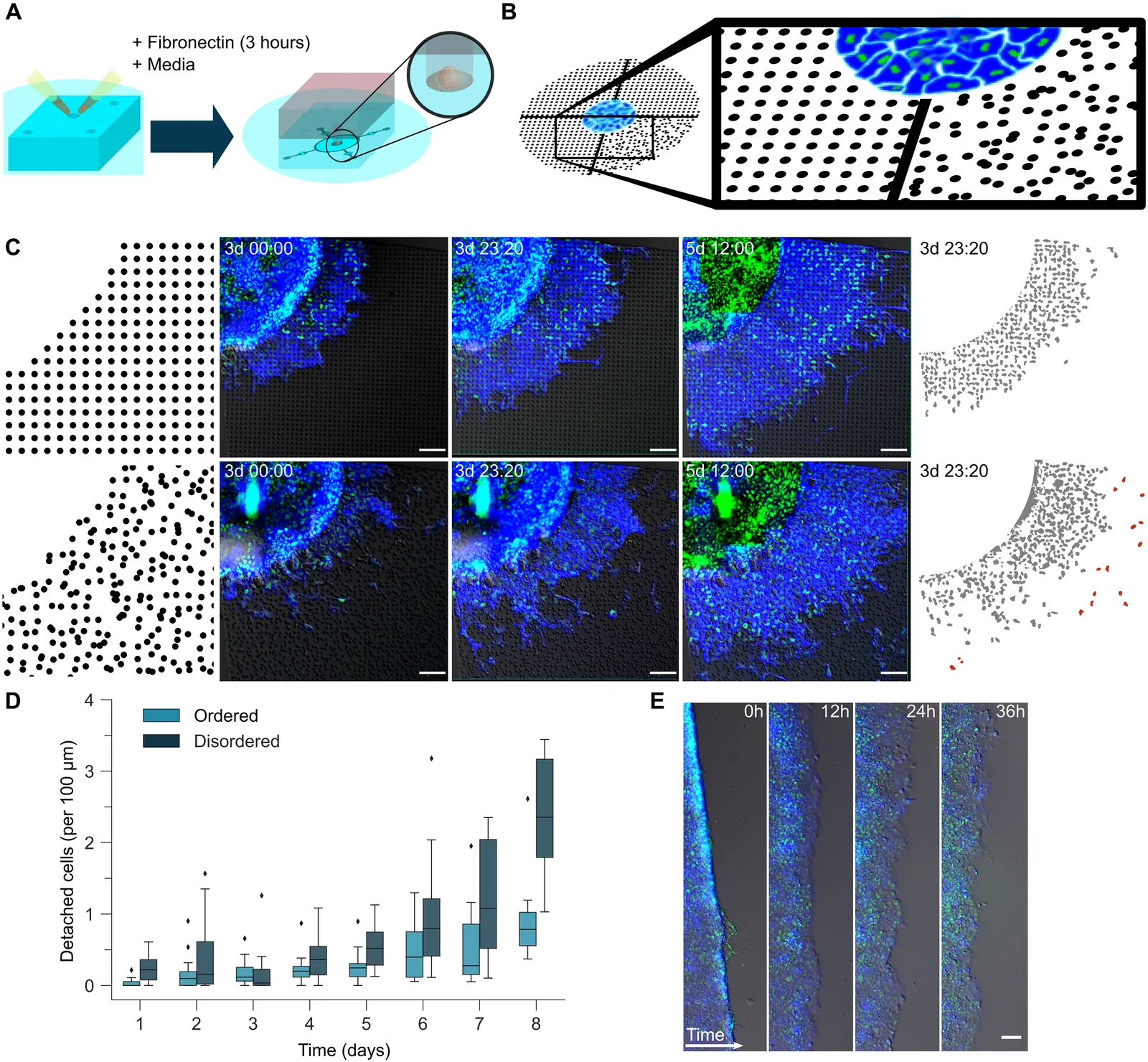
Substrate heterogeneity promotes in vitro tumor cell dispersal. (**A**) Experimental setup of the microfluidic invasion assay. Devices are coated with fibronectin and filled with cell culture media before A431 cells are seeded in the center. (**B**) Illustration of the ordered and disordered pillar patterns used to modulate substrate geometry. Cells invade outward from the central seeding region. (**C**) Spheroid-seeded A431 cell invasion imaged with time-lapse fluorescence microscopy over the days 3, 4, and 5.5 (from left to right). Cells express cytoplasmic mCherry (blue) and nuclear H2B-GFP (green), and cells highlighted in red are detached cells. The right shows segmented nuclei at day 4. (**D**) Number of detached cells normalized by the radial interface length in the respective pillar patterns for cells A431 WT (spheroid-seeded). Mann-Whitney *U*: ordered versus disordered, *P* = 0.0075. Boxplots show median, quartiles, and 1.5 interquartile range; *N* = 16 for ordered, *N* = 18 for disordered environments, followed over 8 days. (**E**) Scratch assay in a homogeneous (pillar-free) environment, used as a control for collective migration. Scale bars, 100 μm. These data show that substrate disorder significantly increases the frequency of single-cell detachments, despite identical average pore size and cell type, illustrating a direct role for environmental geometry in driving cell dispersal. d, days; h, hours.

Each microfluidic chip was divided into four sectors, allowing parallel testing of different pillar patterns under identical conditions. The ordered patterns consisted of grid-like arrays of obstacles with constant spacing (Fig. 1A), whereas in disordered patterns, the average spacing was preserved, but individual pillar positions randomly shifted according to a Laplace distribution (Fig. 1B), which was consistent with the human in vivo data, characterized by considerable heterogeneity in pore size in cancerous tissue in particular (fig. S1, B and C). We define the level of spatial disorder by the average displacement of pillars relative to an ordered array (see Materials and Methods). Tracking invasion over 8 days showed that cells invaded ordered patterns with occasional detachments. Notably, in disordered patterns, cells advanced with enhanced single-cell detachments at the invasion front (Mann-Whitney *U* test, *P* = 0.0075; Fig. 1, C and D, and movie S1). This was in stark contrast to experiments on obstacle-free 2D surfaces, where A431 cells remained cohesive with no detachments (Fig. 1E and movie S2).

To further examine the magnitude of the dispersion effect in disordered patterns, we compared wild-type (WT) A431 cells to genetically engineered cells that lacked E- and P- cadherin (EPCad^−/−^). EPCad^−/−^ cells invaded primarily as single cells, irrespective of environmental geometry, as expected (fig. S1, D and E, and movie S3). Notably, WT cells in disordered environments showed comparable detachment frequencies to EPCad^−/−^ cells (fig. S1F). Together, these data demonstrate that substrate disorder alone can drive a drastic shift from collective to single-cell invasion, with an effect that is comparable to genetically inflicted loss of cell-cell adhesion.

Several mechanisms might explain why environmental heterogeneity promotes single-cell detachments, as different geometrical features change in ordered versus disordered patterns. First, we reasoned that disordered pillar patterns contain regions of very narrow pore sizes that mechanically restrict cell-cell contacts. These constrictions could also compress the nucleus, potentially triggering an active cellular response that might propel the cell forward and cause detachment (*39*–*44*). To test this “pore size” hypothesis, we repeated the assay in ordered pillar patterns with smaller spacing (6 μm), forcing nuclei to severely squeeze between obstacles. We found that cells nonetheless invaded collectively without increased detachment, arguing against pore size alone as the cause (fig. S2A).

Next, we hypothesized that gradients in pore size in disordered environments could promote detachments. In this “funnel” scenario, cells at the leading edge might pass through a narrow constriction into more open space, while the cell bulk would remain stalled. To test this, we engineered two ordered but graded pillar arrays, in which pore size varied only along the invasion axis; one gradual (sinusoidal design) and one abrupt (stepwise design) (see fig. S2B). Neither of these designs triggered a significant increase in detachments (Mann-Whitney *U* test, *P* = 0.61 and *P* = 0.88, respectively; fig. S2C). These data show that favorable local obstacle geometry alone is not sufficient to increase cellular detachments. This led us to explore more collective mechanisms, where repeated transitions through heterogeneous pillar configurations accumulate and drive detachment in disordered environments.

### Minimal biophysical simulations recapitulate the effect of environmental heterogeneity

**Fig. 2.**
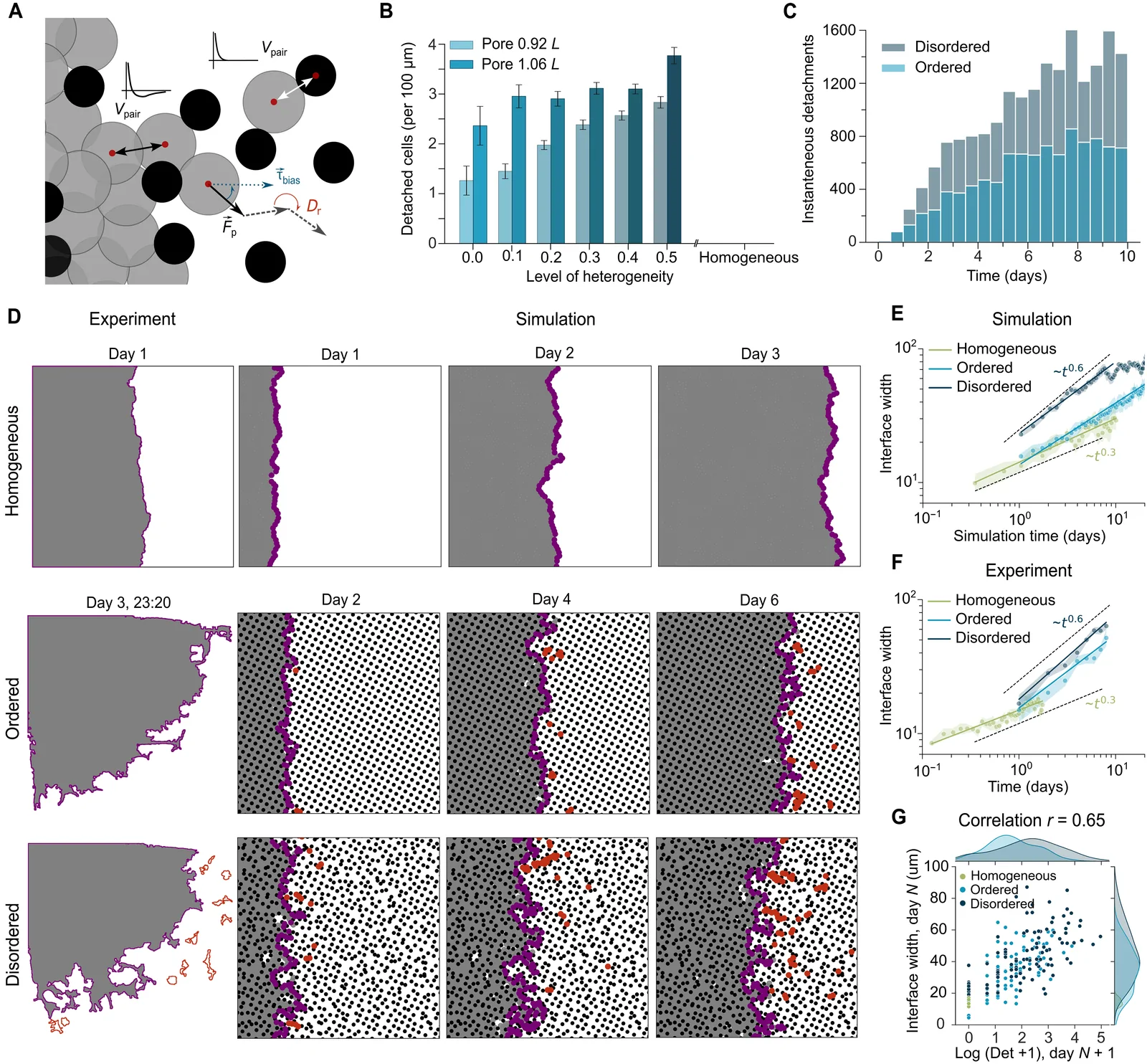
Minimal particle-based simulations reproduce heterogeneity-driven detachments and distinct interfacial growth dynamics. (**A**) Schematic of the simulation setup. Cells are modeled as soft adhesive particles (gray) invading arrays with pillar obstacles (black); relevant forces and interaction potentials are shown. (**B**) Simulated number of detached cells (day 6) as a function of environmental disorder, controlled by the scale parameter of a Laplace distribution (Table 1). Bars show mean ± SEM. OLS slopes: 3.31 ± 0.26 (*R^2^* = 0.98, *P* < 10^−4^) and 2.19 ± 0.51 (*R^2^* = 0.82, *P* = 0.013) for pore sizes 0.92 *L* and 1.06 *L*, respectively. (**C**) Detachment rates increase over time in simulations, more strongly in disordered patterns. OLS slopes: 2.28 ± 0.19 (*R^2^* = 0.89, *P* < 10^−4^) for ordered and 4.29 ± 0.32 (*R^2^* = 0.91, *P* < 10^−4^) for disordered arrays. (**D**) Snapshots of simulated invasion fronts at different time points (magenta), alongside experimental cell interface segmentations [see Fig. 1 (C and D)]. Interface roughness increases over time and with environmental disorder. Detached cells are red. (**E**) Simulated interfacial roughness over time. Power-law fits give growth exponents of β_hom_ = 0.33 ± 0.02, β_ord_ = 0.46 ± 0.01, β_dis_ = 0.53 ± 0.02 (*R^2^* > 0.92, *P* < 10^−4^), consistent with increasing roughening in disordered environments. Markers show means, shaded regions show SEM, and solid lines show linear fits. (**F**) Experimental interfacial roughness measurements yield β_hom_ = 0.25 ± 0.01, β_ord_ = 0.54 ± 0.04, β_dis_ = 0.63 ± 0.04 (weighted least squares, *R^2^* > 0.92, *P* < 10^−4^), showing a good agreement with the theoretical predictions. (**G**) Interface roughness at day *N* strongly correlates with detachments on day *N* + 1 across experiments (*N*_ord_ = 16; *N*_dis_ = 18, *N*_hom_ = 6, Pearson *r* = 0.66, *P* < 10^−4^). Together, these results show that increasing substrate disorder leads to stronger interfacial roughening and elevated detachment rates in both simulations and experiments.

To further test the role of disorder, we turned to in silico simulations of invasion in complex environments. We deliberately adopted a minimal modeling approach, asking whether spatial disorder alone, in the absence of complex cell-intrinsic features, was sufficient to promote cell detachment. Cells were modeled as soft, adhesive, and polar self-propelled particles (*45*–*48*), interacting repulsively with static pillars arranged in different geometries (Fig. 2A). To mirror our analysis of experimental data, detached cells were defined in the simulations as cells separated by distance 1.55 *L* from the bulk, i.e., when the cell-cell contact force becomes negligible (see Materials and Methods for details). Before quantitatively constraining the model parameters, we first performed a broad parameter screen. Despite the model’s simplicity, it robustly recapitulated key qualitative features of the experiments: Detachments increased strongly with the presence of pillars and even more so with pillar heterogeneity (fig. S3A). These features were robust to model variations, for example, adding transient cell-pillar adhesion did not change the phenomenology (fig. S3B).

We then constrained model parameters more precisely, to understand quantitatively what causes detachments in the simulations. As detailed in Materials and Methods [see Table 1 and fig. S4 (A to E) for a summary of the parameter fitting], parameters governing single-cell migration (e.g., migration speed and bias and persistence time) were inferred from the measured behavior of single detached cells in pillar patterns. Cell-cell adhesion strength was constrained on the basis of the lack of detachments in homogeneous environments and infrequent cell-cell rearrangements, consistent with a glass-like rheology (fig. S4F, table S1, and movie S4). Simulations within this parameter regime confirmed that pillar heterogeneity consistently increased detachments [ordinary least squares (OLS) regression, *R^2^* = 0.98 and *R^2^* = 0.82 for pore sizes 0.92 and 1.06 *L*, respectively—see Fig. 2B and movie S5]. Furthermore, reducing adhesion strength not only increased detachments overall but also diminished the additional effect of pillar heterogeneity (*t* test: *P* = 0.47; fig. S4, G and H, and movie S6), in good agreement with experimental results from EPCad^−/−^ cells (fig. S1F).

**Table 1.** Summary of the simulation parameters.

Parameter	Notation	Chosen value (simulation units)	Parameter fitting
Friction coefficient	γ	1	Fixed parameter, nondimensionalizes timescales
Attraction cutoff	*r*_cut_	1.55 *L* (1 *L* for cell-pillar interaction)	Fixed parameter (on the order of cell size to avoid next-neighbor interactions)
Self-propulsion speed	*v*_0_	0.43 *L/*τ	Fitted to correspond to mean single-cell speed 6.9*10^−3^ μm/s inside the pillar pattern (fig. S4, A and B)
Rotational diffusion	*D*_r_	0.7 τ ^−1^	Fitted according to MSAD of experimental trajectories of detached cells (fig. S4C)
Bias torque	*k*_bias_	0.75 rad/τ	Fitted according to the drift of experimental trajectories of detached cells (fig. S4D)
Cell fluidity number	γσ*v*_0_*/*ε	0.52	Constrained by cell confluency while allowing occasional bulk cell rearrangements (fig. S4F, movie S4, and table S1)
Pore size	*L*_p_	0.88–1.3 *L*	Free parameter (on the order of cell size; lower bound ensures that cells can pass through pores individually)
Pore heterogeneity	*H*	μ = *b =* 0.1*L*_p_–0.5*L*_p_	Defined by parameters of Laplace distribution; upper bound taken from disordered patterns in experiments

With the constrained parametrization, we ran many repetitions of simulations with a specific disordered pattern and calculated the probability distribution of detachment as a function of position within the pillar pattern. This analysis, based on clustering instantaneous cell positions and tracking both transient and long-term detachments, revealed that detachment probability was most notably correlated with time, rather than a specific local position. The frequency of detachments consistently increased (OLS regression: *R^2^* = 0.9, *P* < 10^−4^) as the invasion progressed, with much steeper increase in disordered patterns (Fig. 2C).

### Substrate heterogeneity drives KPZq-type roughening of tumor invasion fronts

From a physical perspective, a classical result on the invasion dynamics of a fluid material such as a tissue is that its interface becomes progressively rougher over time, following a highly universal trend (*29*, *49*–*52*), which depends only on a few key characteristics of the system. We hypothesized that cell detachments could be connected to these interface properties, with “time-integrated” heterogeneity causing cumulative surface roughening, as each pillar perturbs the advancing front.

To test this, we computed in simulations both the overall interface perimeter (which increased continuously during cell invasion; fig. S5A) and the surface roughness (i.e., deviations in interface height from its mean; Fig. 2, D and E). The interface roughness always increased in time, but the growth exponent depended strongly on geometry. Power-law fits yielded β = 0.33 ± 0.02 in homogeneous environments—consistent with KPZ dynamics, a highly universal model of interfacial growth. Notably, disordered patterns showed markedly faster roughening with β = 0.5 to 0.6. This is close to predictions for the KPZq universality class (*53*–*56*), which describes interfaces in the presence of quenched noise (Fig. 2E). Given that pillars are static in our simulations, they can be considered as quenched noise sources for the migrating interface.

Computing β across a wide parameter space (table S2) confirmed that these exponents were robust despite the additional features absent from continuum KPZ models (e.g., adhesion and self-propulsion). Furthermore, as a computational control, we performed sensitivity analysis of interface extraction (fig. S5B), and although the previous analysis is based solely on the connected interface, we also extracted the exponent β via an alternative mechanism, from the coarse-grained profiles of particle density in the direction of invasion, including detached cells (*51*), which gave similar results (fig. S5C). To further test the identification of the universality classes, we independently analyzed the height-height correlation function $C\left(r\right)=\langle {\left[h\left(x+r\right)-h\left(x\right)\right]}^{2}\rangle ∼r^{2\alpha }$ from the simulations at late times, to extract the roughness exponent α (*57*, *58*). In homogeneous environments, α = 0.54 ± 0.03, again consistent with KPZ scaling (α ≈ 0.5; fig. S5D). In disordered environments, α increased to 0.71 ± 0.01, which is in agreement with the KPZq universality class (*58*). Last, we asked whether the measured exponents jointly satisfy the Family-Vicsek scaling relation (*49*), which predicts that data from different lateral system sizes collapse onto a universal curve when roughness and time are rescaled as *w*/*L*^α^ and *t*/*L*^*z*^, respectively. Using the independently extracted exponents (α ≈ 0.55, β ≈ 0.33 in homogeneous and α ≈ 0.70, β ≈ 0.55 in disordered environments), we observed a very good collapse across system sizes (fig. S5E). This stringent scaling test (*59*) provides strong evidence that environmental disorder fundamentally alters the universality class of collective cell invasion.

Notably, computing surface roughness and the growth exponent β in the experimental data revealed strong agreement with the simulations (β = 0.25 ± 0.01 in homogeneous and β = 0.63 ± 0.04 in disordered environments; Fig. 2F), which was robust to interfacial extraction methodology (fig. S5F). In simulations with ordered pillar patterns, we found that the predicted growth exponent depended both on the average pore size and the orientation of the pattern relative to invasion direction (table S2 and fig. S6, A and B). However, the ordered environments in simulations always yielded intermediate growth exponents (β = 0.4 to 0.5), with consistently lower interface width than in disordered environments. This trend was confirmed experimentally—ordered patterns exhibited β = 0.54 ± 0.04, and the average interface width was 25% lower compared to disordered environments (Mann-Whitney *U* test, *P* = 0.0004).

Having rationalized the dynamics of interfacial growth, we next asked whether this could explain the different trends of temporal increase in detachments across conditions. To test this, we correlated interface roughness at day *N* with the number of detachments at day *N* + 1 in each experimental replicate. We found a strong correlation (Pearson *r* = 0.66, *P* < 10^−4^) across all conditions, suggesting that there might be a mechanistic link between interfacial roughening and cell detachments (Fig. 2G). To further test this idea, we revisited geometries that did not produce detachments, such as ordered patterns with small pores and sinusoid/stepwise designs (fig. S6, C and D, and movie S7). Simulations with these pillar geometries reproduced the experimental outcome—both interfacial roughness and number of detachments remained lower than in disordered environments (fig. S6E). Together, these results indicate that substrate disorder promotes detachments by enhancing interface roughening, motivating us to examine the concrete mechanism underlying this link.

### Quantitative local and global determinants of cellular detachments

**Fig. 3.**
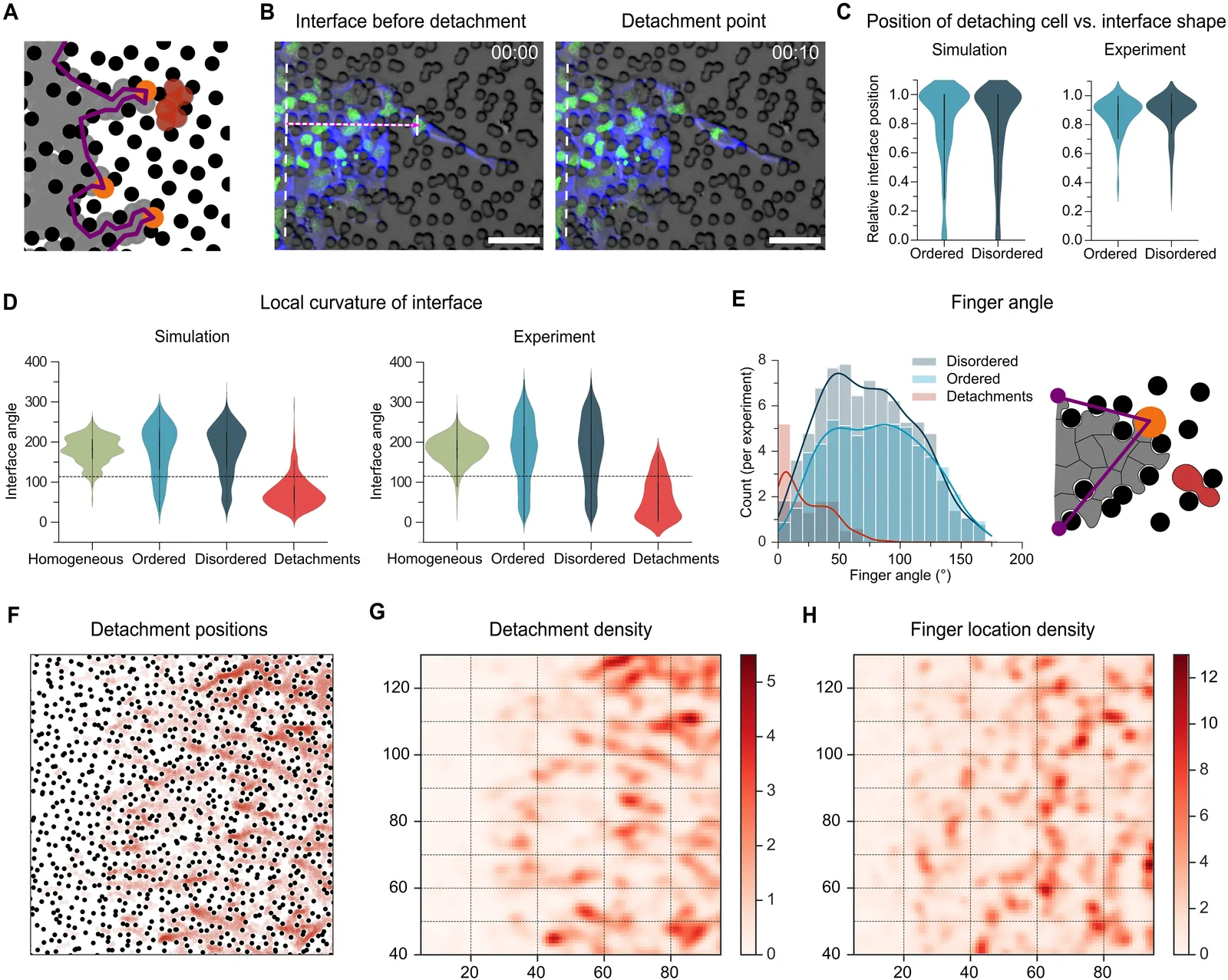
Local interface geometry predicts cell detachment across conditions. (**A**) Interface shape analysis in simulations. Cells are gray, pillars are black, protrusion tips are orange, and detached cells are red. (**B**) Schematic for measuring detachment position relative to interface shape in experiments. Vertical dashed line corresponds to base of the protrusion, and horizontal line measures the distance of base to the protrusion tip. Cells express cytoplasmic mCherry (blue) and nuclear H2B-GFP (green). Scale bars, 100 μm. (**C**) Distribution of detachment positions relative to interface shape in simulations and experiments. Detachments occur predominantly at protrusion tips across all conditions. Detachment position is normalized between the base (0) and tip (1) of the nearest protrusion. The last time frame before detachment is used. Violin plots show the data distribution, with the median, quartiles, and 1.5 interquartile range indicated (*N* = 100 detaching cells from *N* = 7 experiments; *N* > 700 cells from *N* = 40 simulations per condition). (**D**) Local curvature distributions of interface in homogeneous, ordered, and disordered environments, compared with curvature at detachment sites. Pillar patterns, particularly disordered ones, contain more high-curvature regions. Horizontal line marks the threshold curvature, i.e., where probability density of detachment events exceeds that of nondetaching interfaces (113° and 115°, respectively; see Materials and Methods and fig. S7A). Experiments: *N*_ord_ = 16; *N*_dis_ = 18; *N*_hom_ = 6; Simulations: *N* = 40 per condition. (**E**) Experimental protrusion aspect ratios, defined by the angle between protrusion tips and adjacent local minima (“shoulders”). Disordered environments generate narrower, more elongated protrusions (ordered versus disordered, *P* < 10^−4^). Same dataset as in (D). (**F** to **H**) Spatial distribution of interfacial protrusions (H) and detachments (G) across 10 simulations using the same disordered pillar pattern (F) shows that the protrusion locations strongly predict detachment sites (Pearson *r* = 0.6, *P* < 10^−4^). These results show that sharp and convex interface protrusions strongly predict detachment events in simulations and experiments.

To follow up on this finding, we asked how a global property such as interface roughness connects to the local occurrence of cell detachments. We hypothesized that rougher fronts generate interface regions with higher curvature and fewer cell contacts, which might facilitate detachments (*60*–*62*). Analysis of individual detachment events in both simulation and experiment showed that the cells were most likely to detach from the tip of convex, “finger-like” protrusions (Fig. 3, A to C). We next analyzed the local curvature of the interface immediately before detachment, which is inversely related to the number of neighbors (Fig. 3D). Most detachments occurred from interface positions above a threshold curvature value. By contrast, cells migrating in pillar-free environments never reached such high interfacial curvature values, quantitatively explaining the absence of detachment in this condition. To make this criterion more quantitative, we defined a curvature threshold consistent across all geometries (see Materials and Methods for details), which provided strong predictive power. It shows consistent values in simulations and experiments (113° and 115°) and explains 86 and 85% of the detachments in simulations and experiments, respectively (Fig. 3D and fig. S7A). Although the shape of curvature distributions of ordered and disordered patterns appears similar in experiments, likely due to the challenges of segmenting very rough interfaces (fig. S7, B and C), we found that the disordered patterns yield 50% more sharper protrusions than ordered ones (finger angle < 50°, *P* < 10^−4^), especially at late times (Fig. 3E and fig. S7D). Our theory was further supported by a strong spatial relationship between detachments and interfacial protrusions created by the heterogeneous environment (Pearson *r* = 0.6, *P* < 10^−4^; Fig. 3, F to H, and fig. S7E). Together, these findings support the idea that cell dispersion in pillar patterns is a cumulative phenomenon. The cell front invades with a gradual increase of interfacial roughness, with geometry-dependent scaling exponents. The existence of a roughness threshold which is permissive for detachments explains that detachments do not occur at early times in the pillar patterns and never in the pillar-free conditions (within our experimental window).

**Fig. 4.**
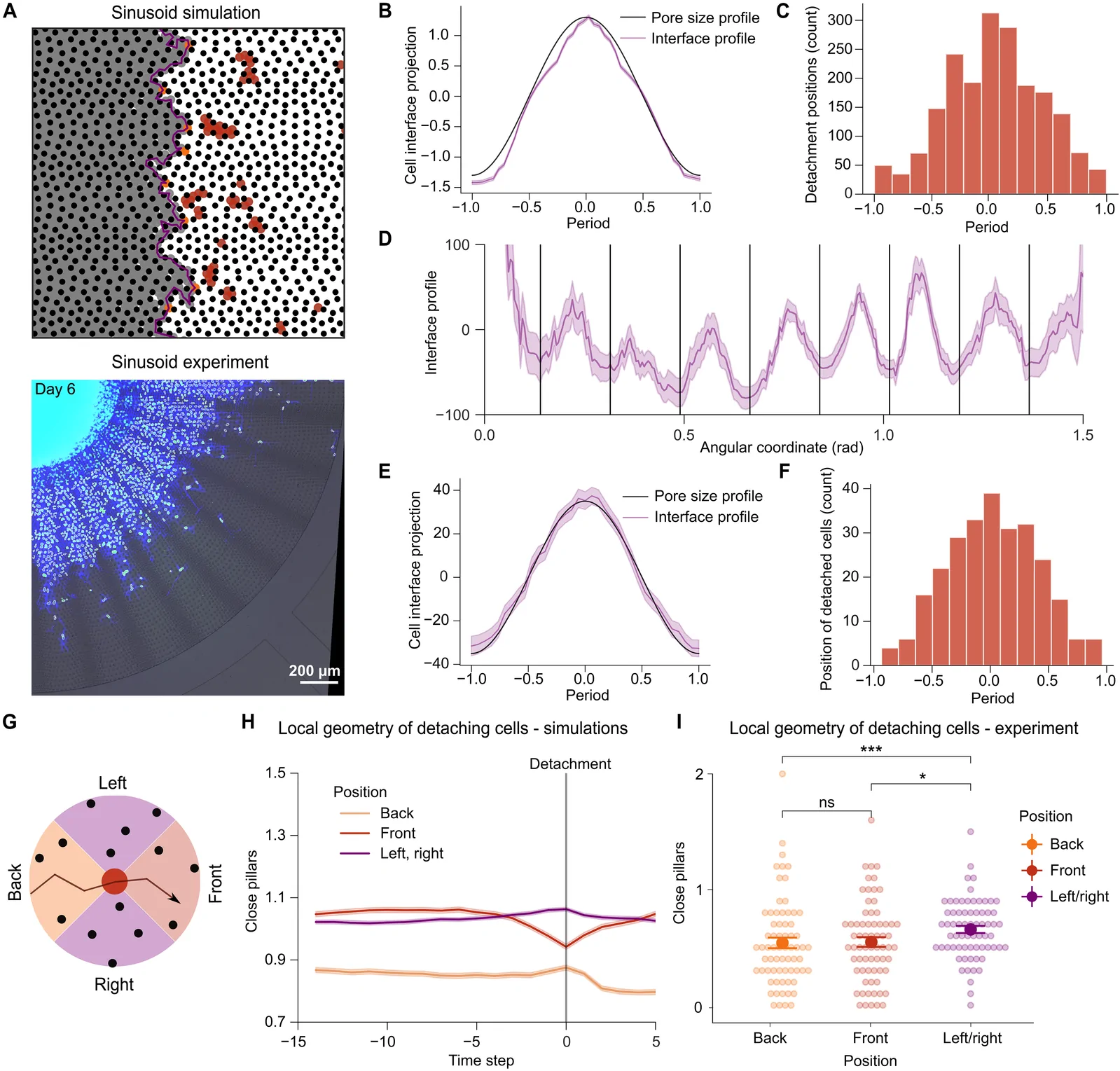
Predicting detachment statistics from global and local heterogeneity. (**A**) Simulations (top) and experiments (bottom) with a longitudinal sinusoidal pillar pattern with periodic variation in lateral pore size. Cells invade with forming regularly spaced finger-like protrusions. Simulated cells are gray, pillars are black, protrusion tips are orange, cell interface is magenta, and detached cells are red. In the spheroid-seeded experiment, cells express cytoplasmic mCherry (blue) and nuclear H2B-GFP (green). (**B**) Simulated interface profile averaged over all time points and sinusoidal periods (*N* = 10), showing protrusions aligned with lower pillar density regions (mean ± SEM). (**C**) Spatial distribution of simulated detachment events showing preferential detachment in low-density regions. (**D**) Experimental interface profile in sinusoidal geometry, averaged over time points and plotted in spherical coordinates (*N* = 4). (**E** and **F**) Average interface profile (E, purple) and positions of recently detached cells (F, red bars), showing agreement with simulation predictions. (**G**) Schematic for quantifying local anisotropy in pillar geometry using four quadrants around each spatial location (front, back, left, and right). (**H**) In simulations, cells approaching detachment experience decreased front-facing pillar density and increased side-facing density, indicating that detachments occur in locally anisotropic regions (time = 0 at detachment, *N* > 10,000 detachment events from 40 simulations; mean ± SEM). (**I**) Experimental measurements of local pillar density at detachment (*N* = 68 detachment events from seven experiments). Cells detach more often in regions with increased side confinement, as simulations predicted. Dot plot shows mean ± SEM. Wilcoxon test: back versus left/right, *P* = 0.0045; front versus left/right, *P* = 0.018; back versus front, *P* = 0.74. Together, these results show that both global pattern–induced interface shaping and local anisotropic confinement jointly predict detachment sites at the invading front. ns, not significant; *P ≤ 0.05; ***P ≤ 0.001.

To further test this mechanism via independent experiments, we designed pillar geometries consisting of sinusoidal patterns oriented perpendicular to the invasion axis. By making the sinusoidal modulation relatively mild, we ensured invasion across the entire pillar pattern while systematically controlling curvature modulation (Fig. 4A and movie S8). Our simulations predicted that this geometry promotes formation of periodic finger-like protrusions at regions of maximal pore width (Fig. 4B) and increases detachment probability with the frequency of sinusoidal modulation, by producing sharper fingers (fig. S7F). In both simulations and experiments, the detachment probability scaled with the local interface shape, confirming that modulating the interface curvature alone is sufficient to increase the number of detachments (Fig. 4, B, C, E, and F).

Last, we asked whether additional local geometrical features predict detachment beyond global integrated heterogeneity, which could be related to the “hotspots” where more detachments occurred in disordered patterns (see Fig. 3F). Since sinusoidal patterns aligned with the invasion axis did not yield detachments (figs. S2, B and C, and S6, D and E), whereas perpendicular sinusoidal patterns did (Fig. 4, A to C), we quantified metrics of local pillar anisotropy relative to the invasion direction (front, back, and sides; Fig. 4G and fig. S7, G and H). In simulations, regions with locally anisotropic free space (i.e., fewer pillars parallel to invasion compared to perpendicular) displayed a small but significant correlation with detachments (*R^2^* = 0.1, *P* < 10^−4^). When combined with the long-term trend of roughness-induced increases in detachment, predictive power increased substantially (*R^2^* = 0.64, *P* < 10^−4^; fig. S7G). Individual simulated cell trajectories consistently showed increased pillar anisotropy specifically at detachment events (Fig. 4H). To test this experimentally, we quantified local pillar anisotropy across *N* = 68 detaching cell trajectories. We found significantly more pillars oriented perpendicular than parallel to the invasion direction at the time of detachment (Fig. 4I). Together, these results demonstrate that disordered environments promote detachments primarily via globally increased interfacial roughening and finger-like protrusions but that local substrate anisotropy further modulates the likelihood of cell detachment.

## DISCUSSION

Our results demonstrate that environmental heterogeneity is an important driver of cancer cell dissemination and show that the geometry of the collective invasion front strongly affects the detachment events. We find that substrate heterogeneity promotes detachments from the bulk with two complementary mechanisms. First, as cells invade disordered environments via active migration, the interface gets gradually rougher, effectively integrating heterogeneity over time. This appears to be a generic effect, since the roughening dynamics map onto the KPZq universality class of moving interfaces. After a threshold level of roughness is reached, cells at the tips of protrusions have few enough neighbors to detach from the collective. Second, this global permissive effect is compounded by local substrate geometry, where anisotropic pillar arrangements increase detachment probability at specific locations. Disorder therefore influences invasion both globally and locally, providing a robust physical framework that may generalize to other collective cell systems beyond cancer.

This provides a path toward cellular dispersion that is complementary to the classical model of epithelial-mesenchymal transition (*63*) via down-regulation of cell-cell adhesion. We expect our proposed detachment mechanism via interface curvature modulation to be relevant as long as cell-cell adhesion remains strong. Consistent with the idea that the threshold curvature for detachment is driven by the strength of adhesion to neighbors, we found that detachments in EP-Cad^−/−^ cells are not only more numerous but can also occur further away from the tip of interfacial protrusions (fig. S8, A and B), as predicted by the model (fig. S6A). Furthermore, while our experiments were in a controlled in vitro setting, where environmental geometry can be finely tuned, the next step would be to relate our observations to more realistic in vivo situations (*64*). For example, different organs have different architectures of ECM (*65*), which fall between fully ordered and fully disordered states, although this spectrum has not been systematically quantified. Preliminary experiments using spheroid invasions in 3D collagen matrix suggested a similar two-step process of interface roughening, followed by detachments (fig. S8C and movie S9), although precise and systematic control of ECM architecture is not possible in 3D (*66*). Extending our simulations to 3D fibrous environments could provide in the future new key insights into how tissue organization affects invasion through cross-talk between an environment and a tumor. In line with this idea, quantitative imaging of human tumor samples revealed that spatially disordered or tilted stromal architectures can be negative prognostic factors for clinical outcome (*67*, *68*). Another natural extension of the model is to consider remodeling of the microenvironment via mechanical forces exerted by cell migration (*69*), which could create feedbacks between cells and their microenvironment that further affect invasion dynamics. Last, although quantifications in our system suggest that proliferation is not correlated to local interfacial curvature and thus unlikely to be the cause of interfacial roughness and detachments (fig. S8, D and E), further modeling in other systems could reveal possible effects of feedbacks between migration forces, proliferation, and interfacial properties (*52*, *70*). We anticipate that the combination of microengineered models, minimal simulations, and quantitative pathology has potential to yield a predictive framework for how stromal heterogeneity influences tumor progression.

Using an experimentally controlled bottom-up approach, we introduce stromal heterogeneity as a potential driver of malignant progression. In analogy to organismal evolution, where heterogeneous habitats foster genotypic diversity, a heterogeneous tumor stroma might drive the transformation of tumor cells into different phenotypic states (in our case, the single cell rather than the collective state). Although these phenotypes are transient and nonheritable, they may later become stabilized (*28*) by epigenetic reprogramming and ultimately genetic accommodation. This work thus highlights a potential route by which nongenetic environmental disorder can generate phenotypic diversity that later becomes fixed during tumor evolution.

## MATERIALS AND METHODS

### Experiments

#### Cell culture

A431 cells expressing mCherry were grown in Dulbecco’s modified Eagle’s medium (Gibco, 10569010) containing phenol red, high glucose, GlutaMAX, pyruvate, 10% fetal calf serum (Gibco, 10270106), nonessential amino acids (Gibco, 11140050), penicillin (50 U/ml), and streptomycin (50 μg/ml; Gibco, 15070063) in humidified incubators at 37°C with 5% CO_2_. EPCad^−/−^ A431 cells were made as described by Labernadie *et al.* (*71*). Cells were regularly tested for mycoplasma contamination and preserved in growth medium containing 5% (v/v) dimethyl sulfoxide in cryogenic conditions for longer periods. Cells were transfected with lentivirus containing H2B-GFP construct and selected for blasticidin resistance and then later sorted for GFP signal, regularly.

#### Device fabrication

Patterns were generated with a Python script in SVG format. Designs of photomasks were completed in (Inkscape, v0.92) in layers for each confinement. SVG files were converted to AutoCAD DXF R14 files, further converted to Gerber files (LinkCAD), and lastly printed on chrome photomasks at a resolution of 1 μm (JD Photo Data and Photo Tools). For high-resolution structures, wafers were first coated with a submicrometer layer of SU-8 as a sticky substrate for later SU-8 structures. We used GM-1040 SU-8 photoresist (Gersteltec) at 5000 rpm for 2 min with flood ultraviolet (UV) exposure. The feature layer was then applied with SU8-2005 (Microchem). According to the datasheet, a UV-absorbing glass plate and higher exposure were used to make sharp features. PL-360-LP from Omega Optical and 550 mJ/cm^2^ with an EVG mercury lamp-based mask aligner gave good results in the 3- to 9-μm thickness range. The thick (25 μm) loading channels were made with Microchem SU8-3025 using the EVG mask aligner according to the Microchem data sheet without the PL-360-LP plate. The master wafers underwent vapor silanization for 1 hour with (1H, 1H, 2H, 2H-perfluorooctyl) trichlorosilane. The final results were single hydrophobic master wafers containing several aligned layers. Polydimethylsiloxane (PDMS) devices were made with the Sylgard 184 Elastomer Kit (Dow Corning) using a ratio of 10:1. The PDMS was mixed in a Thinky mixing machine and then poured on the wafer in a 3D printed tray and further degassed several times in a vacuum chamber. The devices were baked at 80°C overnight. The hardened devices were carefully peeled off the mold and cut into single devices using a razor blade. Holes were punched with a custom arbor press or with skin biopsy puncture tools (1 mm) for the central hole, and sticky tape was used to remove debris. Glass coverslips were sonicated for 5 min in 100% isopropanol and blown dry. Devices were bonded to the glass slides by oxygen plasma and then sealed together at 85°C for 1 hour to make the bond permanent.

#### Invasion assays

Microfabricated devices were inserted into 12-well cell culture plates from the bottom side and glued with molten paraffin. Devices were filled with fibronectin (F1141, Sigma-Aldrich) solution [diluted in 1:5 from stock solution (1 mg/ml) in phosphate-buffered saline (PBS)] using a syringe pump, sealed, and incubated for 3 hours in cell culture growth conditions. Devices were later flushed with 100 μl of cell culture growth medium with syringe pumps. Unless specified otherwise, spheroids of cancer cells (generated with hanging drops) were dropped to sink. If cell suspension was specified, then a previously medium filled 4- to 5-mm cut pipette tip was placed on the center hole. Subsequently, cell suspension of 2000 cells in 1.5 μl of the cell culture medium was introduced into the tip. Cells were left sink passively into the center hole. Cells were incubated until they sank into the hole and imaged at later days. Wells were checked for contamination under a microscope.

#### 2D spread assay

A431 cells were mixed with cytoplasmic mCherry reporter expressing A431 cells (10:1) and assembled into 1000 cell spheroids in hanging drops overnight. Glass coverslips were coated with fibronectin [F1141, Sigma-Aldrich, diluted in 1:40 from stock solution (1 mg/ml) in PBS] for 1 hour and then washed with PBS. Subsequently, chambers were filled with cell culture media, and spheroids were placed to adhere on the substrate. Cells were observed with fluorescence and differential interference contrast (DIC) microscopy.

#### Scratch assay

To observe invasion on a nonconfined 2D surface with an initial straight interface, cells in suspension were placed and grown to full confluence on fibronectin-coated glass substrates (see the “2D spread assay” section above). Then, approximately at the center of the coverslip, cells were scraped with a rubber cell scraper, and detached cells were aspirated and washed away with cell culture media. Cells were observed with fluorescence and DIC microscopy.

#### Imaging

Cells were either imaged once a day at room conditions or in cell culture conditions for time-lapse imaging (10-min interval) for several days (typically 3 to 4 days). Invasion was recorded with an inverted wide-field Nikon Eclipse Ti-2 microscope equipped with a Lumencor light source (475 nm for GFP and 594 nm for mCherry) and an incubation chamber, heated stage and CO_2_ mixer (Ibidi). In general, the fluorescence imaging was accompanied with light microscopy (DIC) imaging. For long-term imaging, phenol red color was checked, and the temperature was monitored with a temperature sensor. Custom made CNC milled box and a pair of 3D printed plugs were used to prevent CO_2_ and humidity leak.

#### Collagen assay

A collagen mixture was created with a final collagen concentration of 1.7 mg/ml by mixing bovine collagen (PureCol, Advanced BioMatrix) in 1× minimum essential Eagle (Sigma-Aldrich) and 0.4% sodium bicarbonate solution (Sigma-Aldrich). Mixture was checked for a pale pink color to ensure for correct pH for polymerization. Twenty µl of the collagen mixture was mixed with 9 μl of the growth medium and mixed gently. Subsequently, 1 μl of the spheroid containing growth medium was added to the mixture and mixed by stirring with the tip. Final mixture was gently placed on a glass coverslip, which was previously glued under a 60-mm dish with molten paraffin. The dish was flipped to let the collagen drop hang from the glass coverslip, whereas the plastic lid was filled with droplets of PBS to keep the dish humidified inside the incubator. Collagen drop was polymerized in the incubator for 45 to 60 min under cell culture conditions. Before imaging, PBS was removed, the dish was flipped again, and the medium was added to the chamber with the collagen drops. Invasion into the collagen was imaged with light microscopy (DIC) over several days under cell culture conditions.

#### Lentivirus production

Lentivirus production was performed as described previously (*72*). Briefly, LX-293 human embryonic kidney cells (Clontech) were cotransfected with LentiCRISPRv1 or LentiCRISPRv2, packaging-ps-PAX2 (Addgene no. 12260) and pCMV-VSV-G envelope plasmids using Lipofectamine 2000 (Thermo Fisher Scientific) as recommended by the manufacturer, and cells were resuspended in the growth medium 1 day before transfection. The supernatant was collected after 72 hours and stored at −80°C.

### Image processing and experimental analysis

#### Image analysis

Fiji was used for image processing, including using the TrackMate plugin for manual cell tracking (*73*, *74*). Proprietary ND2 files from Nikon NIS Elements were converted using the Bio-Formats plugin (*75*) with the physical calibration (pixel size and imaging interval). Images were aligned and processed into TIFF and HDF files. Most of the analysis processes were automated with ImageJ macros. Cell nuclei (H2B-GFP, green) and the remaining cell body (mCherry, blue) and nuclear H2B-GFP (green) were initially segmented using Ilastik, and then particle analysis feature was used to quantify invasion (*76*). Cells were tracked semiautomatically with TrackMate, and tracks were corrected manually. Dividing and dying cells were excluded, and all gaps were filled with interpolation. Final images were processed with automated scripts for display and exported into PNG. In images, cells were considered detached when their cell bodies were not considered touching the bulk. Finger measurements were performed manually by measuring the distance from the tip to the finger baseline. Detachments were selected over a wide range of recorded experiments. The pore size of the detaching cell during detachment versus the pore size in front of the cell, which is being detached from, was measured manually with an interactive macro.

#### Experimental data analysis

The cell interface in the experiment was obtained by identifying the nuclei positions of cells at the interface, for better comparison with simulations, which do not model the cell bodies (fig. S7B). Identification of cells at interface was done by first running Delaunay triangulation of all cell nuclei in each time step and subsequently removing the vertices, which cross through the segmented interface of bulk cell bodies (blue line in fig. S7B). This way, we could efficiently find all cells, which are in contact with the very rough and irregular bulk interface. To check the robustness of these interface analysis techniques, we performed the analysis also using cell body outline with 40-μm spacing directly (fig. S5F). Before using the interface positions for analyzing the roughness exponents, we had to remove the overall tilts in the interface present in some experiments (see the left in Fig. 1C and fig. S7C). This was done by removing long-wavelength Fourier modes (>500 μm). The analysis of the local pillar geometry around the detaching cells (Fig. 4I) was performed by identifying all close pillars in a given radius around the *x*,*y* position of the detaching cells and dividing them into four quadrants [front, back, left, and right (see Fig. 4G), the center of the front quadrant being the orientation of the detaching cell]. Multiple radii were tested, all resulting in qualitatively similar results (final value: *r*_max_ = 27 μm, i.e., three times the pore size). The identified pillars in each quadrant were subsequently weighted by their distance from the reference position (*r*_max_ − *r*)/*r*_max_ and summed.

#### Statistics and data visualization

All statistics for experimental data were performed in R (4.0.3) and plotted using the ggplot2 package (R Core Team 2020; Wickham 2016:2). Tracking data were analyzed with TraXpert, an in-house developed particle tracking analysis application. Directionality data were analyzed with the circular package and circular statistics (*77*, *78*). Minor cosmetic adjustments were performed in Inkscape, without affecting the data points or the interpretation of the plots. Figures were assembled in Inkscape and exported into high-resolution lossless PNG images.

#### Gap analysis

US Biomax tissue arrays of normal and cervical SCC BB10011 and CR484 were stained with picrosirius red before image acquisition. Color deconvolution was performed to separate the picrosirius red collagen signal. Regions of interest (240 μm by 240 μm) of normal dermis and cervical SCC were selected and analyzed using the gap analysis tool described by Tozluoğlu *et al.* (*38*), developed in MATLAB. Similar analysis was performed on the templates used to generate the regular and irregular pillars (fig. S1, B and C). Briefly, the deconvolved grayscale image is thresholded into a binary image of ECM. The gap analysis algorithm then works by filling in the gaps between ECM with the largest circle possible until all gaps have been filled in down to a minimum circle diameter of 3 pixels. The frequency distribution of gap radii (in pixels) is recorded alongside gap statistics including mean, SD, skewness, kurtosis, and size percentiles of radii.

### Numerical model

We implemented 2D Brownian dynamics simulations as a minimal model of collective cell migration (*45*–*48*) through complex environments (Fig. 2A). In this framework, each cell is represented as a soft self-propelled particle [approximating its nucleus (*42*)] moving on a substrate. The overdamped equation of motion for each particle is given by

$$\gamma \frac{dr_{i}}{\mathrm{dt}}=\frac{dV_{\text{pair}}}{dr_{i,j}}+F_{p}$$

where γ is the friction coefficient, $r_{i}$ is the particle coordinates, $V_{\text{pair}}$ is the pairwise interaction potential, and $r_{i,j}$ is the distance between particles. The active self-propulsion force $F_{p}$ is given by

$$F_{p}=\gamma v_{0}p_{i}$$

where $v_{0}$ is the intrinsic propulsion speed, and $p_{i}=\left(\text{cos}\theta_{i},\text{sin}\theta_{i}\right)$ defines the orientation of particle i. In the absence of interactions, this force yields an average cell speed of $v_{0}$.

The polarity of migrating cells is subject to rotational diffusion and an external biasing torque $\tau_{\text{bias}}$, which directs cell migration away from the bulk (see below for discussion of its origin). The orientation dynamics are given by

$$\delta \theta_{i}\delta t=\tau_{\text{bias}}+\sqrt{2D_{r}}\eta =-k_{\text{bias}}\text{sin}\left(\theta_{i}\right)+\sqrt{2D_{r}}$$

where $D_{r}$ is the rotational diffusion coefficient, η is a unit-variance Gaussian random variable, and $k_{\text{bias}}$ controls the strength of the bias torque. We define $\theta_{i}=0$ as the invasion direction (to the right in all simulations shown). To validate the numerical implementation, we computed the mean square displacement of a single active Brownian particle without bias torque (*79*). The observed crossover from ballistic to diffusive motion was consistent with the used value of $D_{r}$.

To describe cell-cell interactions, we assume a simple pairwise potential consisting of short-range repulsion and intermediate-range attraction, as previously described (*45*, *46*, *80*). The potential takes the form

$$V_{\text{pair}}=\sum_{i}\sum_{j>i}v_{\text{pair}}\left(r_{i,j}\right)$$

$$v_{\text{pair}}\left(r_{i,j}\right)=\left\{\begin{array}{cc} 4\epsilon \left[{\left(\frac{\sigma }{r_{i,j}}\right)}^{4}-{\left(\frac{\sigma }{r_{i,j}}\right)}^{2}\right]+\Delta v_{\text{pair}}\left(r_{i,j}\right), & r_{i,j}<r_{\text{cut}}\\ 0, & r_{i,j}\geq r_{\text{cut}} \end{array}\right.$$

where $r_{i,j}$ is the distance between particles *i* and *j,* ϵ is the depth of the potential well, σ is the characteristic interaction distance, and $r_{\mathrm{cut}}$ is the cutoff distance. The potential is shifted by a linear correction term $\Delta v_{\mathrm{pair}}$, ensuring that both the potential and its derivative smoothly vanish at the cutoff $\frac{dv_{\text{pair}}}{\mathrm{dr}}\left(r_{\text{cut}}\right)=0$.

To model the structured environments (pillar geometries), we introduced stationary particles into the simulation box that contribute to the pair potential. The cell-pillar interaction was cut off at its minimum ($r_{\mathrm{cut}}=\sqrt{2}\sigma$) so that only repulsive interactions remained, as there is no experimental evidence for stable cell-pillar adhesion. A key assumption of our minimal modeling approach is that intrinsic cellular parameters (e.g., motility and adhesion strength) are kept constant. Differences in detachment dynamics observed between conditions are thus entirely attributed to changes in substrate geometry.

#### Details of the simulation setup

We used the HOOMD-blue v3 library to perform numerical simulations (*81*). The equations of motion were integrated using the Euler-Maruyama scheme with a time step of dt=0.001 τ. All simulations were conducted in two dimensions.

Simulation parameters and results are reported in dimensionless units. For convenient comparison with experiments, lengths are expressed in units of cell-cell equilibrium distance *L* (defined as the interaction minimum $1L=\sqrt{2}\sigma \approx 16\;\mu m$) and time in units of $\tau =10^{3}\;s$. Simulation output was saved every 600 frames, corresponding to the smallest experimental time step (10 min). Unless stated otherwise, the simulation parameters used in the model are summarized in Table 1.

Before doing a systematic parameter search, we first constrained parameters to reasonable ranges and conducted nondimensional analysis to identify the key dimensionless ratios. The fluidity of a tissue in the absence of obstacles is governed by a dimensionless number relating self-propulsion to cell-cell interaction strength ($v_{0}\sigma \gamma /\epsilon$) (*47*, *82*, *83*). If the ratio is too low, cells behave as a solid, with very few neighbor rearrangements. Conversely, above a certain threshold, the tissue fluidizes, as motility-driven rearrangements become possible. However, when considering not only bulk tissue but also cell interfaces, very large values of this ratio can lead to spontaneous cell detachments in pillar-free environments, resembling a gas (*84*). Experiments with monolayers invading a homogeneous environment support an intermediate value of this ratio, as we observed virtually no detachments, while cells remained able to move relative to one another (Fig. 1D, movie S4, and table S1).

Moreover, we used the presence of single detached cells in pillar experiments to constrain several parameters related to single-cell migratory properties. Precisely, by calculating instantaneous (*x, y*) displacements and the mean squared angular displacement (MSAD) (*85*) of detached cells in both simulations and experiments, we were able to constrain the values of self-propulsion speed (*v*_0_) and particle persistence length (*v*_0_/*D*_r_) (fig. S4, A to C). The value of external bias (*k*_bias_) was fixed by analyzing the radial drift in trajectories of detached cells in experiment and comparing it to simulations (fig. S4D). We detected strong biased migration even in single cells far from the leading edge, indicating that this bias does not arise simply from pushing forces due to bulk proliferation. Given previous reports of self-generated gradients guiding long-range migration (*86*, *87*), such mechanisms are compatible with this bias, although we chose to remain agnostic about its origin in the simulations. Last, we performed one further sanity check by examining the mean squared displacement (MSD) of single detached cells in simulations and experiments, allowing us to validate the chosen combination of parameters *D*_r_, *v*_0_*,* and *k*_bias_*.* The final parameter set reproduced the experimental MSD curve most closely, yielding the highest coefficient of determination (pseudo-*R^2^* = 0.89), particularly at long times (fig. S4E).

The simulations of migrating cells used for comparative statistics across various pillar geometries and parameters (Fig. 2B; figs. S3, A and B, and S4, and table S2) were initialized in a rectangular box of size *Lx* = *Ly* = 425 *L*. The left portion of the box was filled with cells in hexagonal packing, while the right side contained the pillar geometry. Simulations used for close temporal comparison with experiments (Figs. 2, C to E, 3, and 4, and figs. S5 and S6) were initiated in a smaller box (*Lx* = 425 *L*, *Ly* = 106 *L*) with new cells added on the left side during the simulation to maintain a stable bulk pressure. Each parameter combination was run with at least 10 repeats. The ordered pillar geometry was constructed as a square lattice, with pillar spacing being twice the effective pillar diameter. Reported results for ordered pattern simulations (e.g., Figs. 2, B, C, and E and 3D) are averaged over four orientations of the square lattice (0°, 15°, 30°, and 45°; fig. S5A). The disordered pattern was generated from the ordered patterns of all four tilts by shifting each pillar by a random angle and distance following a Laplace distribution from its original position. The fully disordered pattern corresponded to Laplace distribution parameters μ = *b =* 0.5 *L*_p_.

#### Simulation analysis

The initial time required for cells to accumulate at the front of the pillar pattern and enter it was excluded from the analysis. Unless stated otherwise, simulation frames were analyzed at a frequency of 6 τ after the start of the invasion. Simulations were run until bulk cells reached the end of the pillar pattern. All simulation analyses were performed in Python.

The overall number of detached cells (Fig. 2, B and D, and figs. S3, A and B, S4G, and S6D) was determined by distance-based clustering of cells, using *r*_cut_ as the natural clustering cutoff from a physical perspective, and summing the number of cells within the pattern that were not connected to the bulk at a given time point. The identification of pattern locations with high detachment probability and instantaneous detachments (Figs. 2C, 3, C, D, F, and G, and 4, C and H, and figs. S4H, S6E, and S7, A and E to G) was performed in two steps. First, the same clustering analysis was applied to each simulation frame. Second, a cell was considered detached if it remained separated from the bulk (largest cluster) for at least five consecutive frames. For each detachment event, we recorded the *x*,*y* coordinates, cell ID, and time step for further analyses, such as relating detaching events to interface properties (Fig. 3, C and D) or local pillar positions (Fig. 4D).

The shape of the cellular interface (e.g., Figs. 2, D and E, 3A, and 4A) was analyzed by first computing the Delaunay triangulation (convex hull) of cellular positions in each simulation frame. The Delaunay triangulation was then filtered using the alpha shape algorithm (*88*) (Python alphashape library) to determine the concave hull of the cellular interface. We performed a sensitivity analysis on the parameter alpha (fig. S5B, final value: alpha = 0.6). The positions of interface cells were subsequently used in the roughness scaling analysis (Fig. 2E and figs. S5, B to E, and S6, B and E) and in the analysis of interface shape, perimeter, and curvature (Figs. 3, A, C, D, and H, and 4B, and fig. S5A). The curvature threshold (Fig. 3D and fig. S7A) was defined as the point at which the probability density of detachment events exceeds that of pillar-free interfaces. The local maxima and minima of the cell interface were identified using the argrelextrema function in SciPy library (*89*). Transient protrusions on the order of cell size (protrusion length = average distance to next local minimum < 1 *L*) were filtered out.

The interface roughness (Fig. 2E and fig. S5, B to E) was quantified using the height function *h(y)*, which represents the level of invasion of cells at different points along the interface (polar coordinates were used for pillar pattern experiments). We analyzed the temporal increase in roughness by computing the interface width *w*(*l,t*), which measures the root mean square deviation of the interface from its mean value, defined as

$$w\left(l,t\right)={\left\{\frac{1}{N}\sum_{i=1}^{N}{\left[(h\left(y,t\right)-\bar{h}\left(t\right)\right]}^{2}\right\}}_{L_{\text{int}}}^{1/2}$$

where *L*_int_ = 1000 μm is the length of the interface contour. These fluctuations grow over time following a power law scaling, $w\left(l,t\right)∼t^{\beta }$, with a characteristic critical growth exponent β (*31*). The growth exponent β was extracted both from the analyzed interface shape (using Delaunay triangulation and alpha shape algorithm) but also via an alternative method, from the coarse-grained profiles of particle density in the direction of invasion, which also included the detached cells (fig. S5C) (*51*).

To quantify the spatial roughness of the invasion front (fig. S5D), we computed the height-height correlation function of the interface profile in simulations. The function is defined as

$$C\left(r\right)=\langle {\left[h\left(x+r\right)-h\left(x\right)\right]}^{2}\rangle$$

where *h(x)* denotes the height of the interface at lateral position 𝑥, and ⟨·⟩ represents an average over all positions 𝑥 and multiple simulation runs. For self-affine interfaces, this function exhibits power-law scaling with distance 𝑟

$$C\left(r\right)∼r^{2\alpha }$$

We computed 𝐶(*r)* from late-time snapshots of the invasion front, when roughness exhibited a statistically stationary regime. The roughness exponent α was subsequently determined by fitting the log-log plot of (𝑟) versus 𝑟 over the intermediate scaling range, avoiding short-range discretization artifacts and long-range saturation effects. We did this analysis for simulations conducted in homogeneous and disordered environments. The resulting values of α provide an independent estimate of the interface roughness exponent, which is complementary to the growth exponent β obtained for time-dependent roughness evolution. For testing Family-Vicsek scaling (fig. S5E), sufficiently long simulations were conducted for multiple lateral system sizes *L*. For each *L*, we ran and averaged ∼20 independent simulations; in homogeneous environments, these differed only by stochastic cell dynamics, whereas in disordered environments, they further differed by independently generated random pillar patterns. We then rescaled the interface roughness and time as *w*/*L*^α^ and 𝑡/*L*^𝑧^, respectively, using independently measured scaling exponents (α = 0.55, β = 0.33 in homogeneous environments; α = 0.70, β = 0.55 in disordered environments), with the dynamic exponent defined as 𝑧 = α/β. The collapse of the rescaled curves across system sizes was taken as evidence that the observed invasion dynamics fall within the corresponding universality class.

The analysis of local pillar heterogeneity (Fig. 4H and fig. S7, G and H) was performed by identifying all neighboring pillars within a radius *r*_max_ = 3.18 *L* (three times average pore size) around a given (*x*,*y*) position in the disordered pattern. The pillars were divided into four quadrants (front, back, left, and right; Fig. 4G). The pillars in each quadrant were weighted by their distance from the reference position using the factor (*r*_max_ − *r*)/*r*_max_, and the weights were summed.
