# Supplementary material for: Substrate heterogeneity promotes cancer cell dissemination through interface roughening
Source: Sci Adv. 2026 Sep 9;12(37):eaed0587. doi: 10.1126/sciadv.aed0587 (PMC13557091; doi:10.1126/sciadv.aed0587)
Supplement: Supplementary file 1 [file sciadv.aed0587_sm.pdf]

Science Advances
AAAS

Supplementary Materials for

Substrate heterogeneity promotes cancer cell dissemination through interface roughening

Zuzana Dunajová *et al.*

Corresponding author: Michael Sixt, michael.sixt@ist.ac.at; Edouard Hannezo, edouard.hannezo@ist.ac.at

Sci. Adv. **12**, eaed0587 (2026)
DOI: 10.1126/sciadv.aed0587

The PDF file includes:

Figs. S1 to S8
Tables S1 and S2
Legends for movies S1 to S9

Other Supplementary Material for this manuscript includes the following:

Movies S1 to S9

Supplementary Materials - Substrate heterogeneity promotes cancer cell dissemination through interface roughening

Supplementary figures and tables

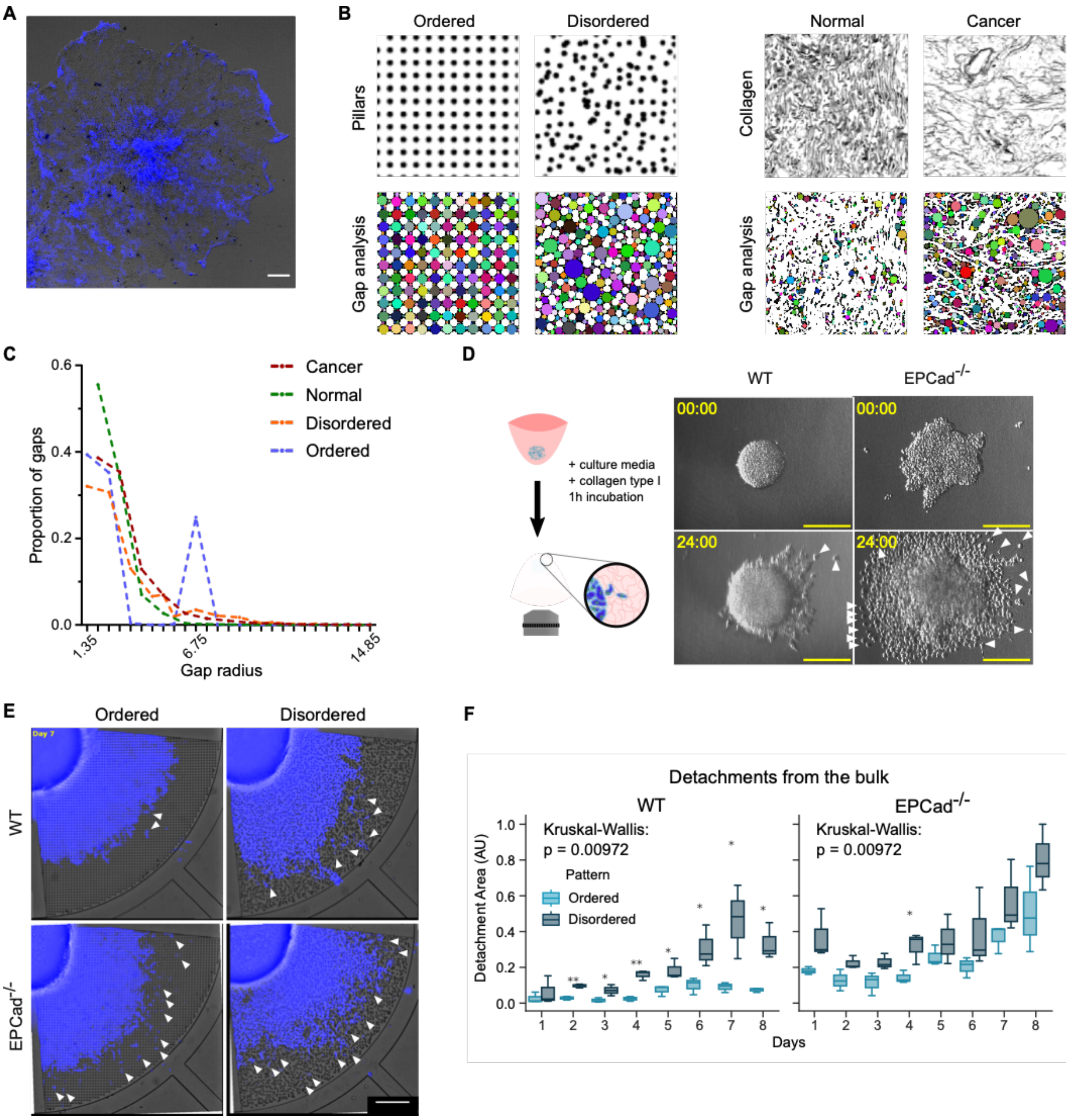

Fig. S1: Invasion of WT and E-,P-Cad$^{-/-}$ cells in different environments. A. 2D invasion assay of WT A431 cells with and without mCherry reporter (1:10 mix). **B-C.** Gap analysis of pillar patterns compared to normal and cancer tissues. Distributions of the gap sizes in cancer tissue closely match those in disordered pillar patterns (*N* = 8 normal and *N* = 15 tumor images). **D.** Collagen invasion assay of WT and E-,P-Cad$^{-/-}$ spheroids imaged by brightfield microscopy. **E.** Detachments of spheroid-seeded WT and E-,P-Cad$^{-/-}$ cells invading ordered and disordered pillar patterns. White arrows indicate detached cells. **F.** Detached cell area in the corresponding pillar patterns for WT and E-,P-Cad$^{-/-}$ cells. Scale bars: 100 µm, boxplots show median, quartiles, and 1.5 interquartile range. *N* = 3 experiments per genotype and pattern followed over 8 days.

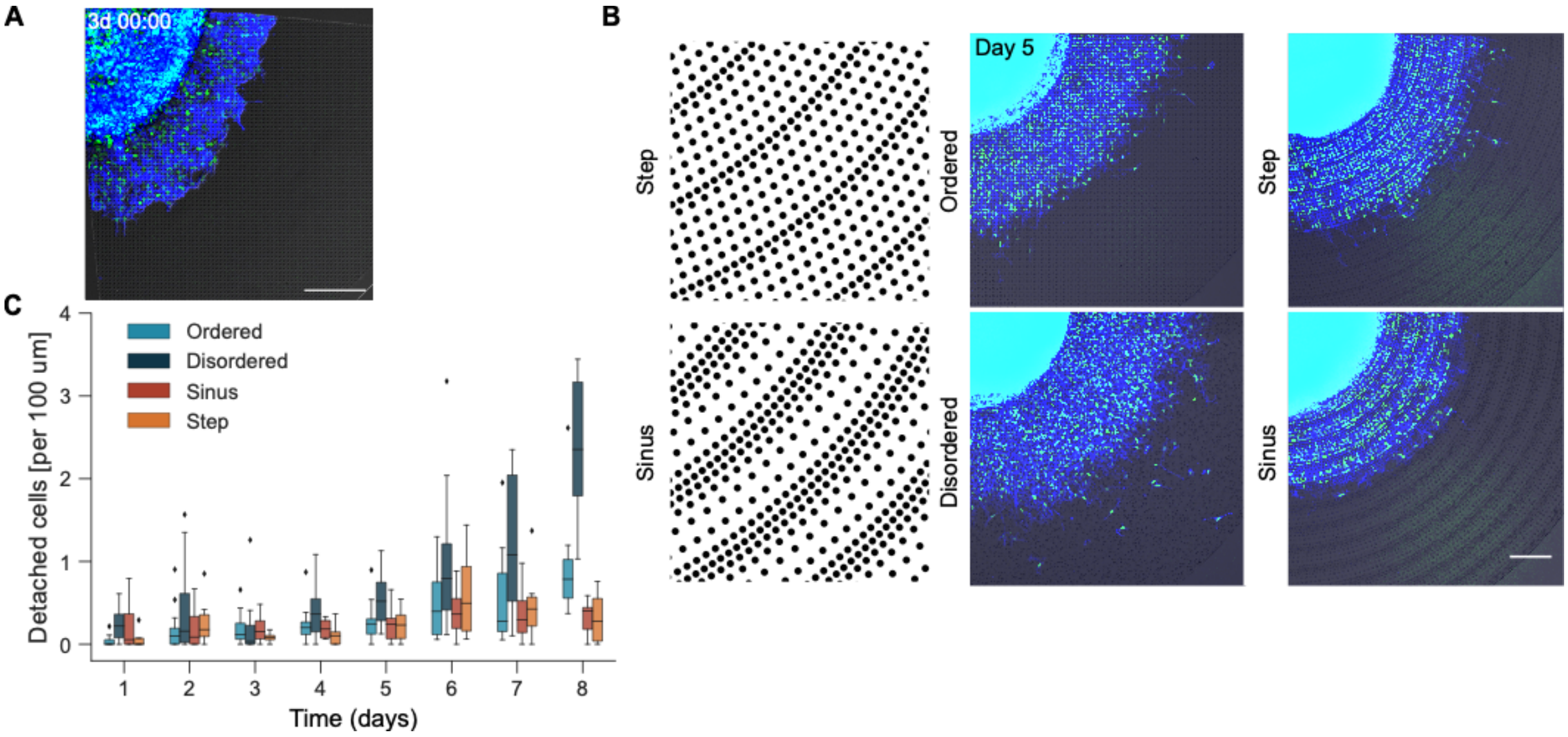

Fig. S2: Invasion dynamics of WT cells in pillar patterns. A. Spheroid-seeded cell invasion assays in ordered pillar pattern with smaller pores (6 µm). **B.** Suspension-seeded cell invasion assays in stepwise or sinusoidal pillar patterns. **C.** Quantified detachments for each pattern. Boxplots show median, quartiles, and 1.5 interquartile range (N_{ord} = 16, N_{dis} = 18, N_{step} = 8, N_{sin} = 8 experiments followed over 8 days). (Mann–Whitney U test: Ordered vs. Sinusoid, p = 0.61; Ordered vs. Stepwise, p = 0.88. Scale bars: 200 µm.

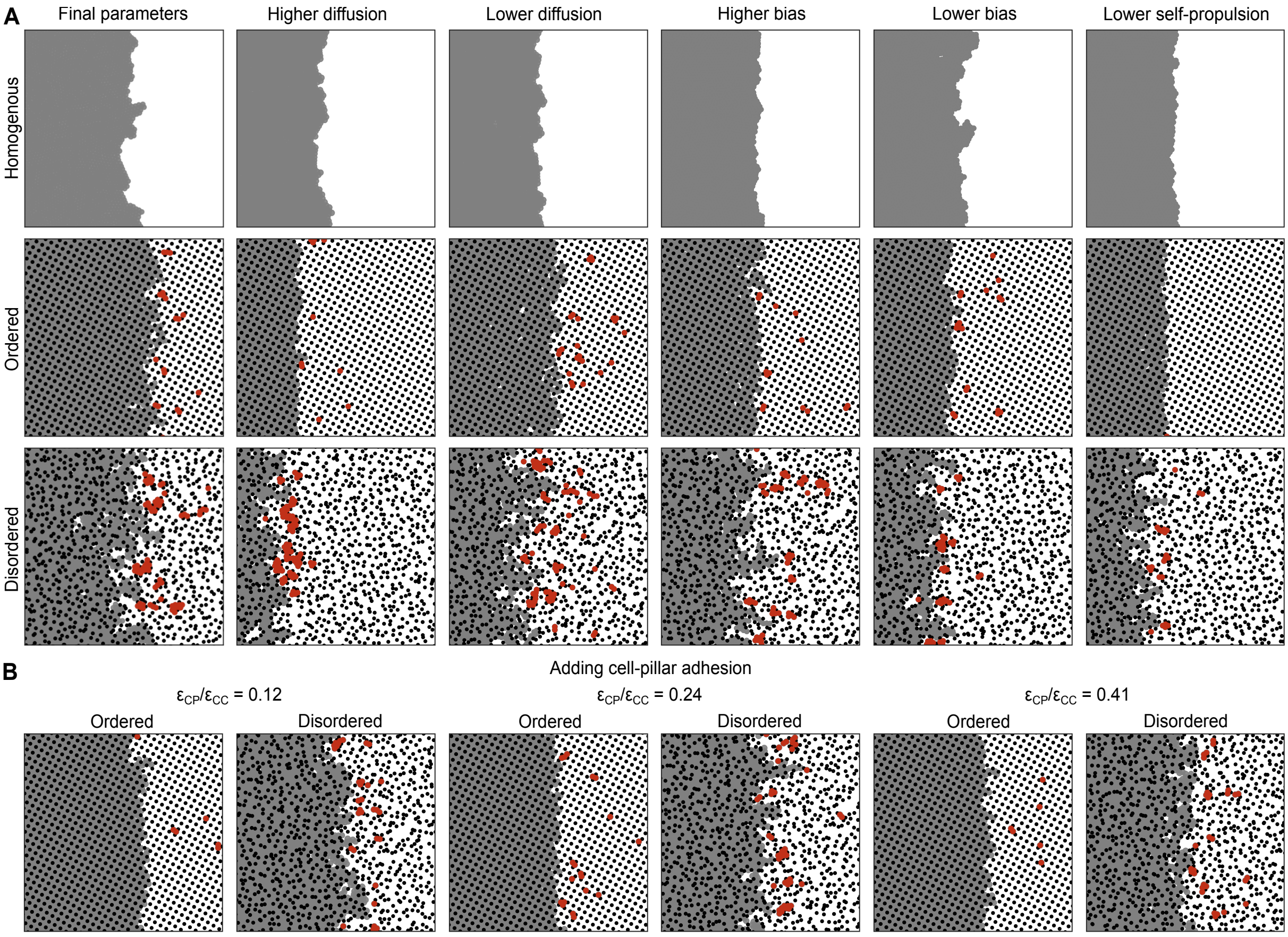

Fig. S3: Phase diagram of invasion dynamics across model parameters. A. Temporal increase in detachment rate occurs robustly in both ordered and disordered pillar patterns across simulation parameters. Snapshots show representative systems at day 6, with the best-fit parameter set used in the main text shown in left column (see Table 1). Subsequent columns show effects of varying individual parameters (D_r = 1 τ^{-1}, D_r = 0.5 τ^{-1}, k_{bias} = 0.5 rad/τ, k_{bias} = 1 rad/τ, v_0 = 0.24 L/τ, respectively). **B.** Simulations of cells in ordered and disordered environments at day 6 for different values of cell-pillar adhesion (r_{cut} = 1.55 L). Cells are grey, pillars black, detached cells red.

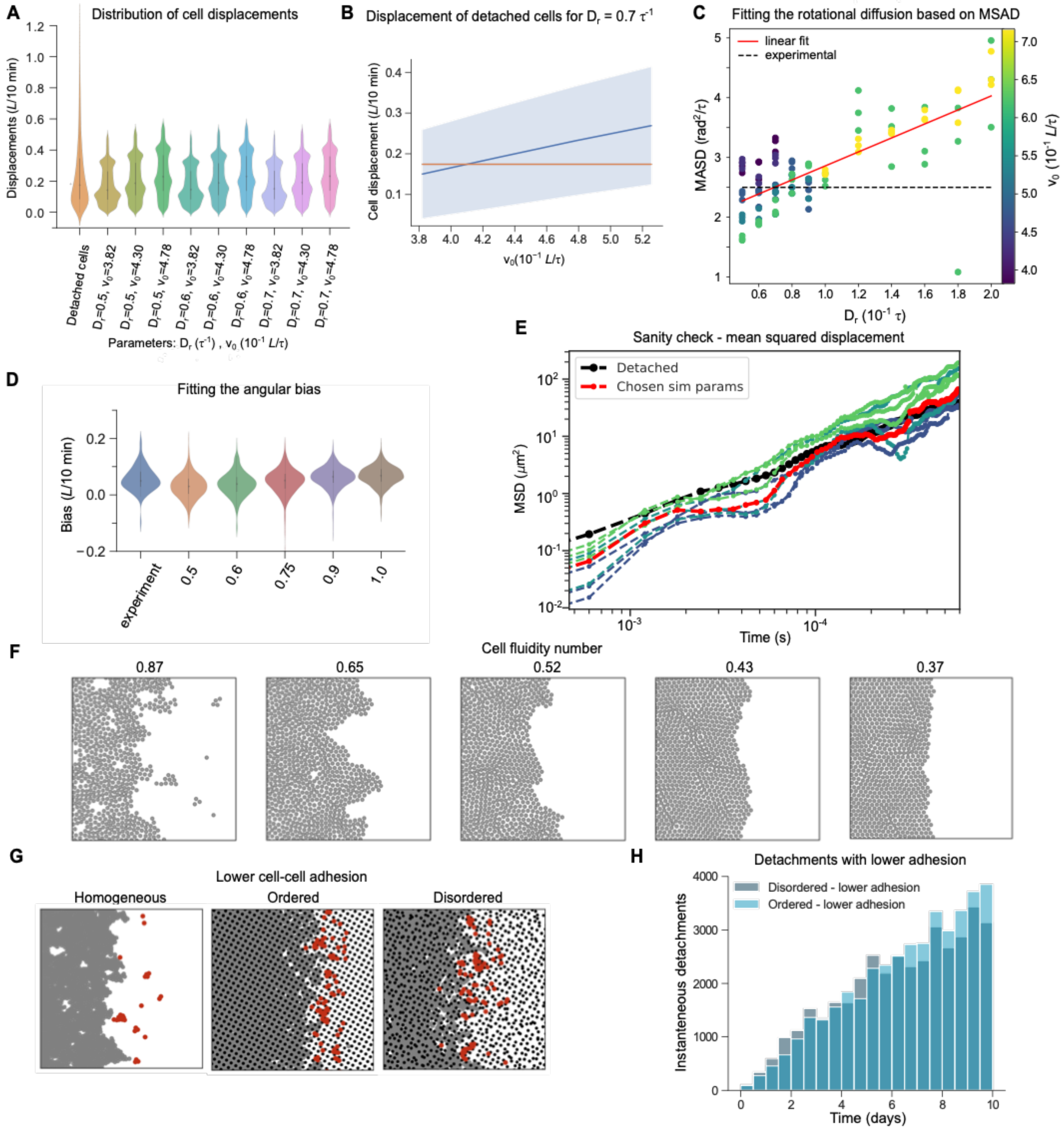

Fig. S4: Model parameter fitting and simulations with reduced adhesion. A-B. Fitting the active force v_0 using displacements of single detached cells in pillar patterns. Experimental data are shown in the first column of A. Simulations (remaining columns; N = 3 simulations of 100 sparsely seeded cells in ordered patterns with pore size 1.06 L) show displacements increase linearly with v_0 and are independent of D_r. By comparing with experiments (orange line in B indicating median of N = 91 trajectories from 6 experiments) we chose $v_0 = 0.43\, L/\tau$ as value used in simulations unless explicitly mentioned. **C.** Fitting the rotational diffusion constant D_r using MSAD of single detached cells in regular pillar patterns. The experimental MSAD (black dashed line) is best reproduced by $D_r = 0.7\ \tau^{-1}$ (linear fit, $R^2 = 0.93$), used in all simulations unless explicitly mentioned. **D.** Fitting k_{bias} from radial bias in trajectories of single detached cells. k_{bias} = 0.75 rad/$\boldsymbol{\tau}$ best matched experimental data (Kolmogorov-Smirnoff test, p = 0.46). **E.** MSD of detached cells comparing experiment and simulations with varying D_r, v_0, and k_{bias}. Parameters from A–D exhibited the highest pseudo-R^2 related to the experimental MSD curve. **F.** Simulation snapshots of cells (grey beads) in homogeneous environments for different fluidity numbers (day 6). High fluidity produced numerous detachments, something not observed in wild-type experiments, whereas low fluidity yielded solid-like rheology without re-arrangements on the time scale of the experiments. **G.** Simulation snapshots of cells with lower cell-cell adhesion, corresponding to E-,P-Cad$^{-/-}$ cells (fluidity 0.87; cells grey, pillar black, detached cells red). Cells detach even without pillars, reducing their environment sensitivity, as seen experimentally. **H.** Quantification of detachments when lowering the cell-cell adhesion in ordered and disordered pillar patterns, showing loss of the ordered-disordered difference (t-test: p = 0.47).

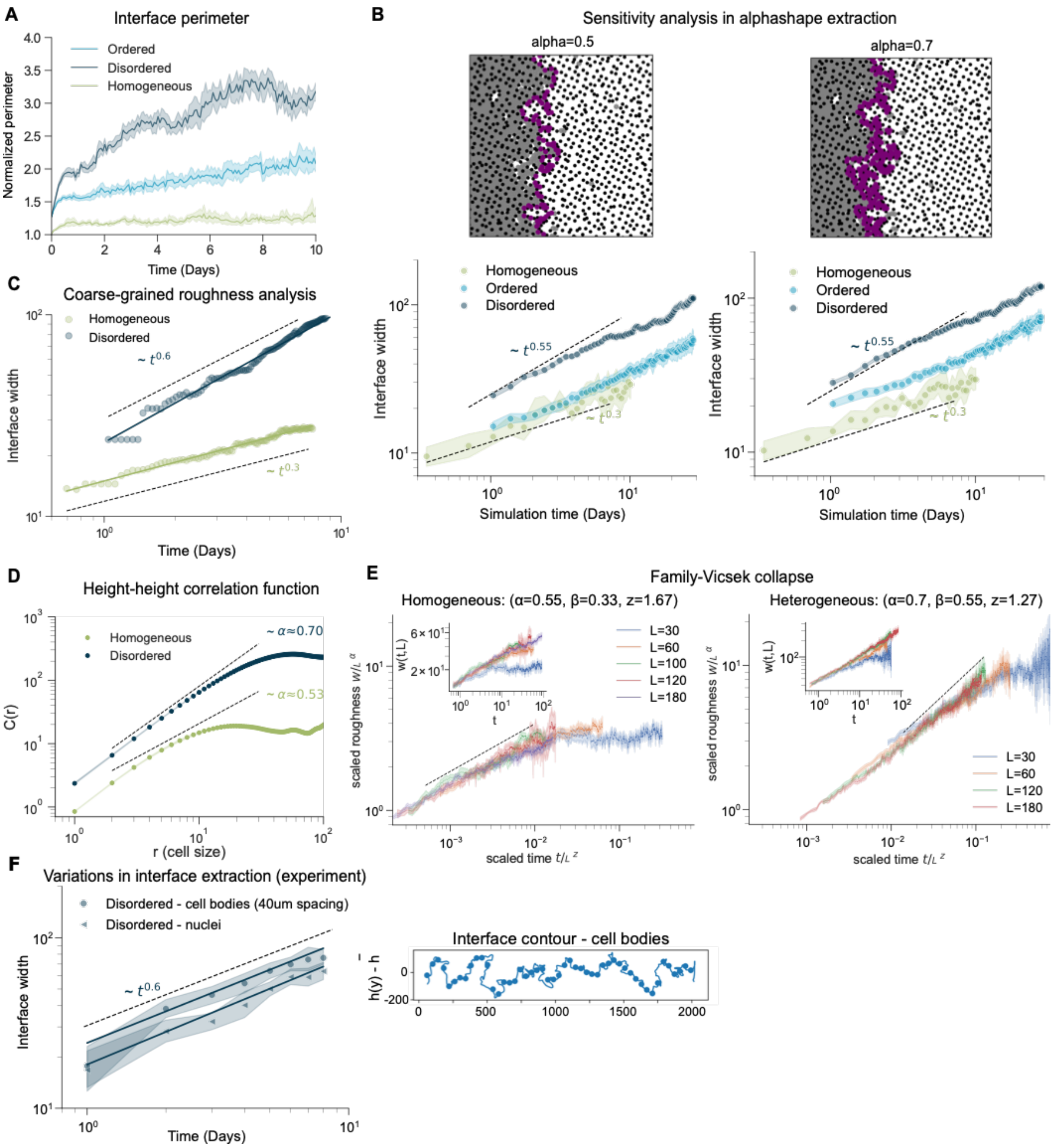

Fig. S5: Universality class identification and interface extraction. A. Temporal evolution of the interface perimeter in simulations (N = 40; mean ± SEM). **B.** Sensitivity analysis of interface extraction in simulations. Top: identified interface cells (magenta) for different values of *alpha* parameter in the alpha shape algorithm. Bottom: corresponding roughness analysis. Changing *alpha* shifts absolute interface width values but does not affect roughness scaling. **C.** Roughness analysis in homogeneous and disordered environments using coarse-grained interfaces based on density profile of all cells. **D.** Height-height correlation function of steady-state invasion fronts in simulations (day 25) used to estimate the roughness exponent α. Shaded regions indicate SEM; fits were performed over the intermediate scaling range (1 < r < 12). **E.** Family–Vicsek scaling collapse of interface roughness in homogeneous (left) and disordered (right) environments. Rescaling according to w/L^{α} versus t/L^{z} collapses curves for different L onto a universal function, consistent with KPZ and KPZq universality classes, respectively. Insets show original curves. **F.** Left: comparison of roughness analysis in disordered environments using two interface extraction methods (cell body outlines with 40 µm spacing and cell nuclei positions). Right: example cell body interface contour and points used for roughness calculations.

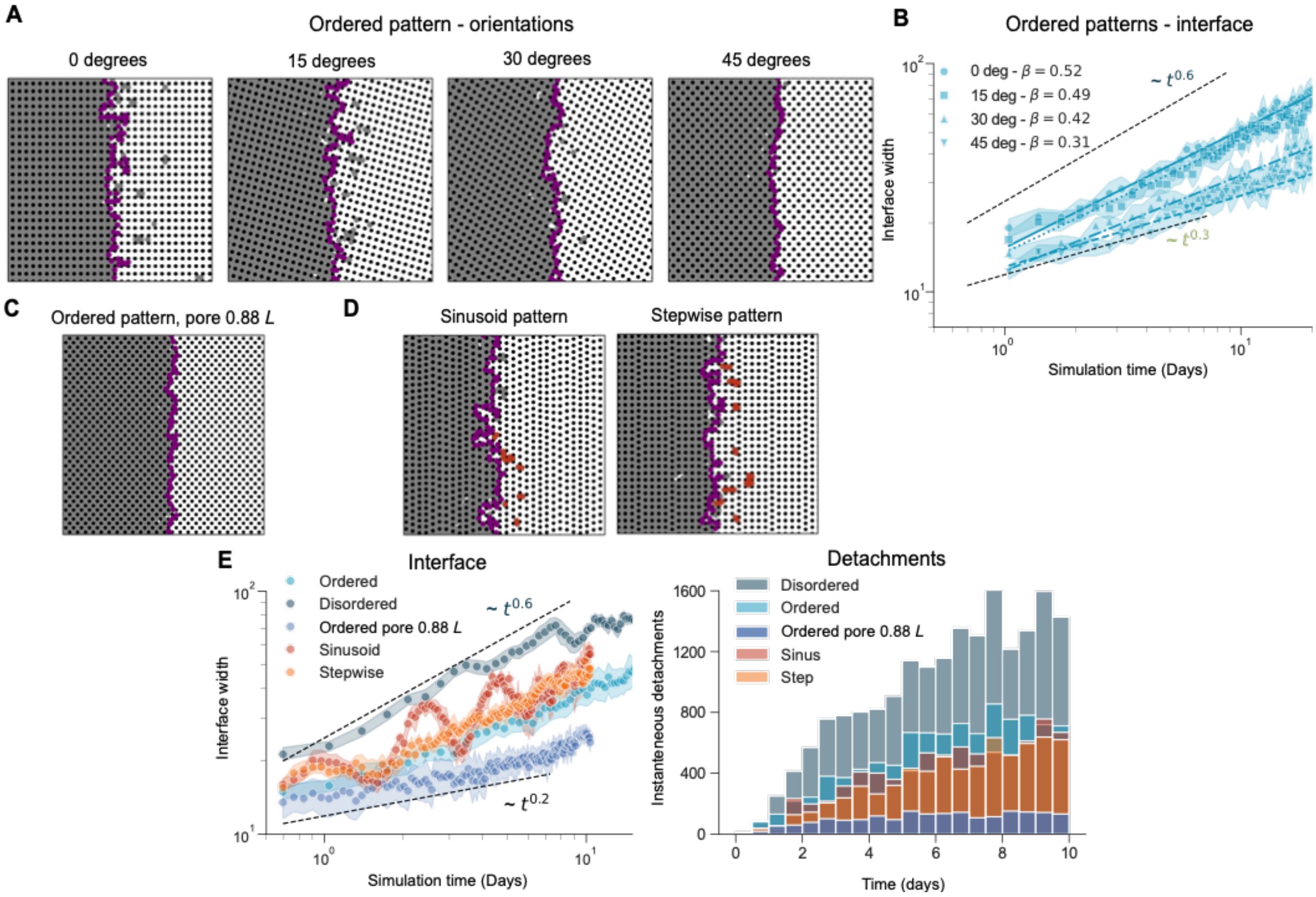

Fig. S6: Simulations with regular, sinusoidal and stepwise patterns. A. Invasion behavior in ordered pillar patterns with different orientations (day 4; cells are grey, pillars black, cell interface magenta). Results from all four orientations were averaged for comparison with other geometries, given that invasion is radial in experiments. **B.** Interfacial roughness and roughness exponents for the four orientations of ordered patterns shown in A (N = 10 simulations each; shaded regions indicate SEM). **C-D.** Simulation snapshots of invasion in ordered pattern with small pore size (6 μm, C), and sinusoidal or stepwise pillar patterns (D). These conditions show lower or comparable detachment frequencies to ordered patterns, consistent with experiments in Fig. S2B-C. Cells are grey, pillars black, detached cells red, and cell interface magenta (day 4). **E.** Quantification of interface width and detachment numbers for simulations in C,D, showing roughness follows the imposed pillar geometry and that some detached cells in stepwise pattern reattach to the bulk, reducing total detachments.

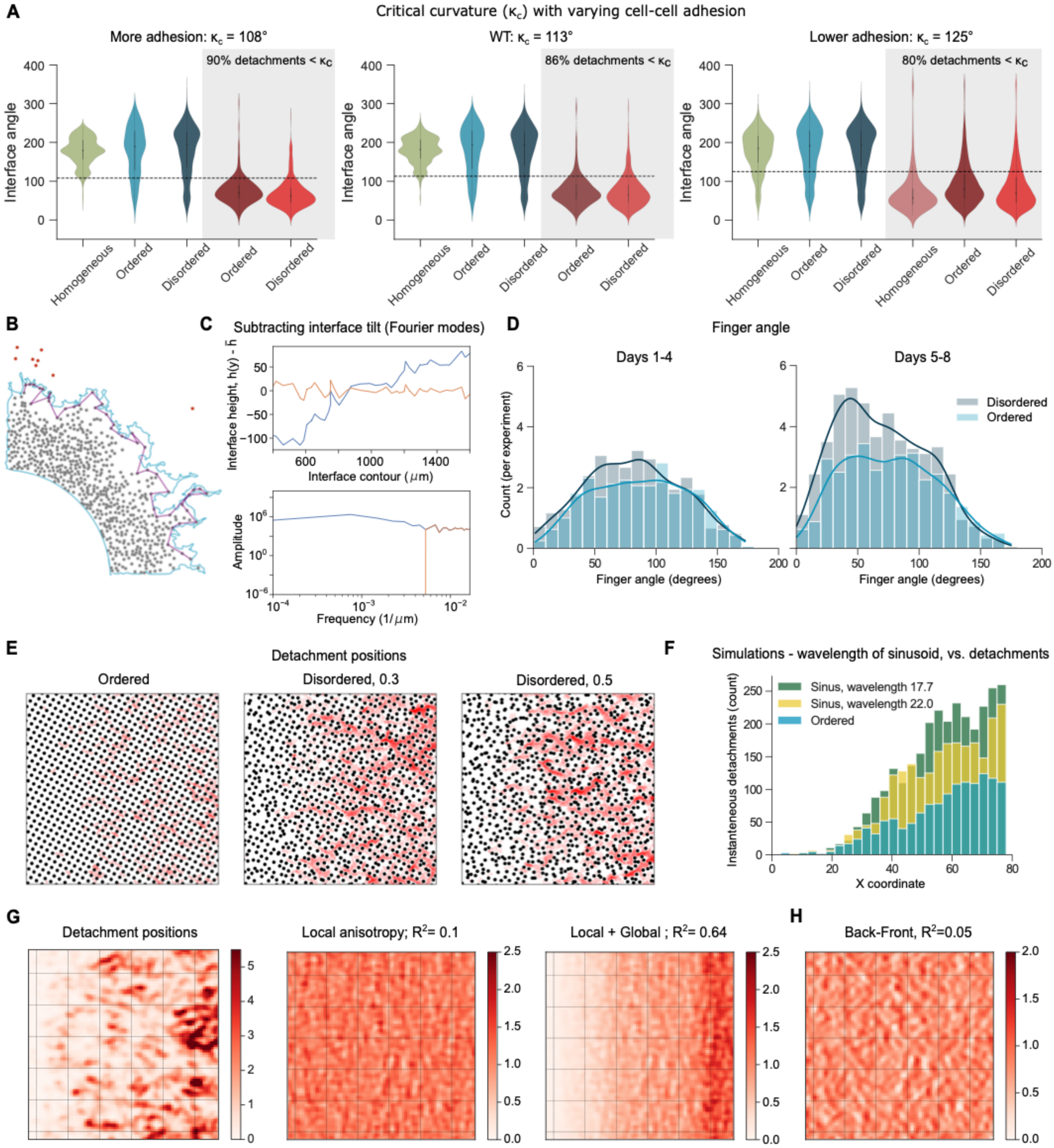

Fig. S7: Detachment statistics and interface curvature in experiments and simulations. **A.** Local curvature distributions of interfaces and prior detachment (grey area) in simulations with varying cell-cell adhesion (fluidity numbers 0.43, 0.52, and 0.87, respectively). Critical curvature (horizontal dashed line) is defined by the intersection of homogeneous and detachment distributions. Detachments occur preferentially at protrusion tips (low angles), although distributions broaden with reduced adhesion. **B.** Experimental interface analysis. The blue line shows segmented cell-body outlines, while the magenta line connects interface nuclei used for roughness and curvature analysis (see Materials & Methods). **C.** Correction of interface tilts (orange) by removing long Fourier modes from the detected experimental interface (blue). **D.** Finger angle distributions in ordered and disordered patterns become increasingly distinct at later timepoints (N = 16 ordered and N = 18 disordered experiments). Smaller angles correspond to sharper fingers (see Fig. 3E). **E.** Spatial distribution of detachment events in simulations with increasing positional disorder in pillar arrays (red indicates higher probability). **F.** Temporal increase in detachments in perpendicular sinusoid simulations with shorter wavelength (see Fig. 4A). **G.** Heat map comparing detachment positions with local pillar anisotropy in disordered patterns. The metric *Left* + *Right* – *Front* shows weak correlation (R^2 = 0.1), whereas including a directional mask, which takes into account the increase of detachments improves prediction (R^2 = 0.63); N = 10 simulations. **H.** Alternative anisotropy metric (*Back* – *Front*), showing weaker spatial correlation with detachments.

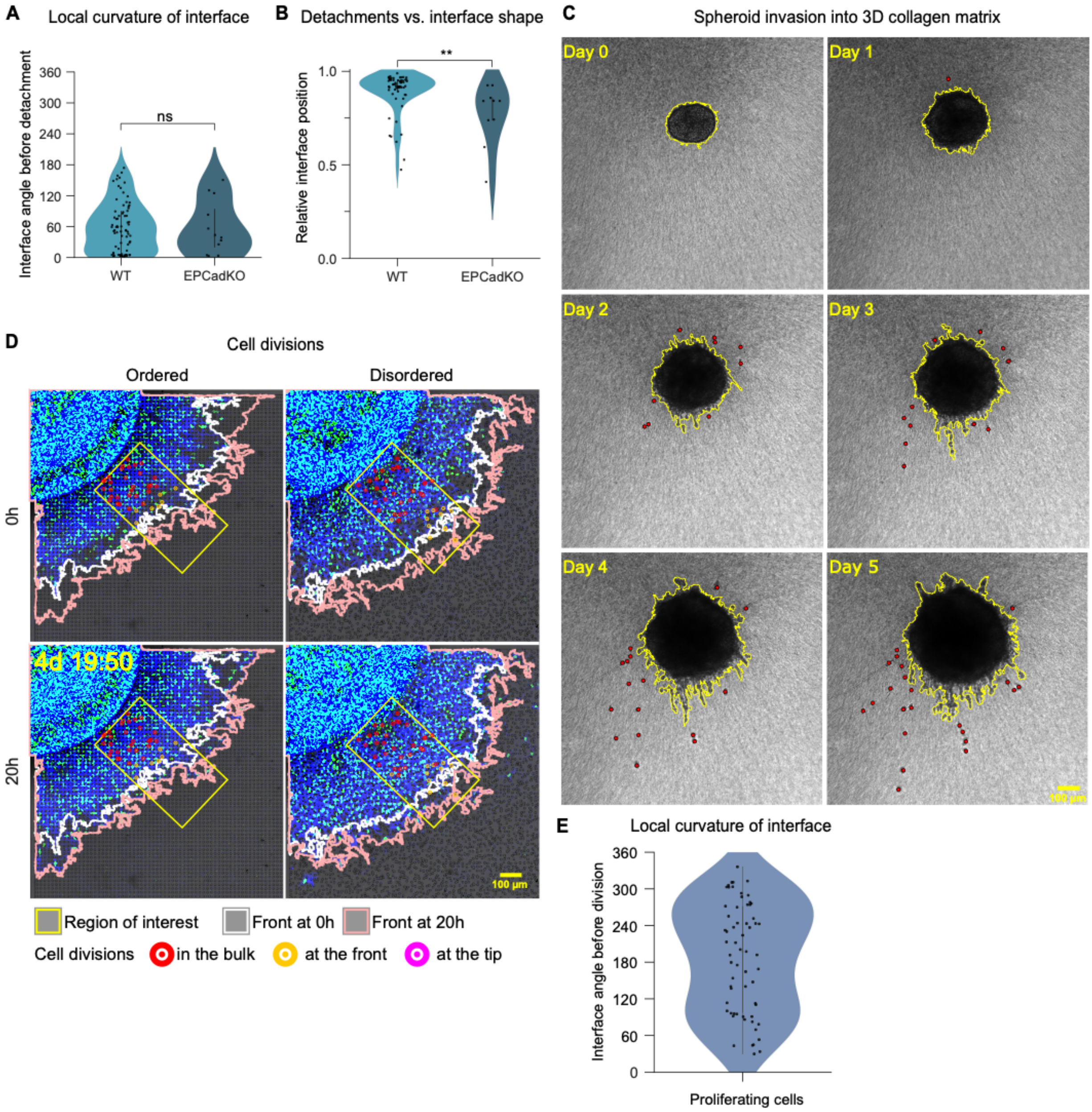

Fig. S8: Analysis of interface geometry, cell division, and 3D invasion. A. Local interface curvature before detachment in WT and EPCad$^{-/-}$ experiments. **B.** Positions of detaching cells relative to interface shape in WT and EPCad$^{-/-}$ experiments. **C.** Spheroid invasion into 3D collagen imaged over 5 days by brightfield microscopy. Interfaces are highlighted in yellow and detached cells in red. Interface roughness increases with formation of finger-like protrusions, while complete detachments occur at later time points. **D.** Visualization of cell divisions during a 20 h imaging window. Dividing cells touching the interface are classified as "front" cells. Both spheroid- and suspension-seeded experiments were analyzed (N_{sph} = 4; N_{susp}=4). **E.** Relationship between front-cell division and local interface curvature before cell division (N_{ord} = 4; N_{dis} = 4). No preferential division was observed in specific interface regions such as protrusions.

Table S1: Cell bulk behavior in homogeneous simulations across parameter sets. Green – KPZ scaling, Yellow – KPZ scaling with holes and detachments, Orange – jammed, collective movement; Grey – jammed, no movement.

D_r [τ^{-1}]	fluidity					
	**0.36**	**0.44**	**0.52**	**0.60**	**0.68**	**0.76**
**0.1**	Orange	Orange	Orange	Orange	Orange	Orange
**0.5**	Orange	Green	Green	Green	Green	Yellow
**1**	Orange	Green	Green	Yellow	Yellow	Yellow
**2**	Orange	Orange	Green	Green	Green	Yellow
**3**	Orange	Orange	Orange	Green	Green	Green
**10**	Grey	Grey	Grey	Grey	Grey	Grey

Table S2: Interface roughness in ordered and disordered patterns across parameter sets. Green – exponents from 0.25-0.44, Blue – larger than 0.55.

Pore size	D_r [τ^{-1}]	Ordered pattern *fluidity*				Disordered pattern *fluidity*			
		**0.44**	**0.52**	**0.60**	**0.76**	**0.44**	**0.52**	**0.60**	**0.76**
**0.88 *L***	**0.5**	0.27	0.33	0.41	0.50	0.55	0.70	0.70	0.57
	**1**	0.19	0.36	0.43	0.44	0.45	0.65	0.72	0.71
	**2**	0.37	0.25	0.23	0.37	0.44	0.36	0.39	0.49
**1.06 *L***	**0.5**	0.48	0.57	0.62	0.59	0.54	0.62	0.78	0.57
	**1**	0.30	0.45	0.52	0.55	0.44	0.69	0.64	0.59
	**2**	0.34	0.23	0.28	0.42	0.35	0.32	0.40	0.43
**1.24 *L***	**0.5**	0.32	0.32	0.38	0.38	0.57	0.66	0.80	0.49
	**1**	0.37	0.46	0.49	0.50	0.58	0.69	0.64	0.59
	**2**	0.33	0.27	0.31	0.41	0.56	0.33	0.40	0.43

Supplementary movie captions

Movie S1: Invasion of A431 human skin carcinoma cells in ordered vs. disordered environments. Cells express cytoplasmic mCherry (blue) and nuclear H2B-GFP (green).

Movie S2: Invasion of A431 human skin carcinoma cells in free environments. Cells express cytoplasmic mCherry (blue) and nuclear H2B-GFP (green).

Movie S3: Invasion of EPCad$^{-/-}$ A431 human skin carcinoma cells in ordered vs. disordered environments. Cells express cytoplasmic mCherry (blue) and nuclear H2B-GFP (green).

Movie S4: Simulations of cells with different fluidity number (i.e. with varying cell-cell interaction strength) in homogeneous environment. Different values of fluidity number exhibit different level of cell rearrangements. Cells are represented as grey beads.

Movie S5: Simulations of cells migrating in different environments (homogeneous, ordered and disordered). Cells are represented as grey and pillars as black beads.

Movie S6: Simulations of EPCad$^{-/-}$ cells (lower cell-cell adhesion) invading different types of environments. Simulations of cells migrating in different environments (homogeneous, ordered and disordered. Cells are represented as grey and pillars as black beads.

Movie S7: Simulations of cell invasion in spatially structured environments. These patterns do not increase detachment rate (regular with smaller pore, sinusoid, stepwise). Cells are represented as grey and pillars as black beads.

Movie S8: Simulation and experiment of cell invasion in sinusoid perpendicular pattern. The cells in the pattern create localized interface protrusions and detachments. Cells in experiment express cytoplasmic mCherry (blue) and nuclear H2B-GFP (green). Cells in simulations are represented as grey and pillars as black beads.

Movie S9: Spheroid invasion into 3D collagen matrix imaged over 5 days with brightfield microscopy. Interface roughness of cells increases with formation of finger-like protrusions, while the complete cell detachments occur in the later timepoints.
